# Supplementary material for: Efficacy of artemisinin-based and quinine-based treatments for uncomplicated falciparum malaria in pregnancy: a protocol for systematic review and individual patient data (IPD) meta-analysis
Source: BMJ Open. 2019 Aug 22;9(8):e027503. doi: 10.1136/bmjopen-2018-027503 (PMC6707703; doi:10.1136/bmjopen-2018-027503)
Supplement: Author's manuscript [file bmjopen-2018-027503.draft_revisions.pdf]

BMJ Open

BMJ Open is committed to open peer review. As part of this commitment we make the peer review history of every article we publish publicly available.

When an article is published we post the peer reviewers' comments and the authors' responses online. We also post the versions of the paper that were used during peer review. These are the versions that the peer review comments apply to.

The versions of the paper that follow are the versions that were submitted during the peer review process. They are not the versions of record or the final published versions. They should not be cited or distributed as the published version of this manuscript.

BMJ Open is an open access journal and the full, final, typeset and author-corrected version of record of the manuscript is available on our site with no access controls, subscription charges or pay-per-view fees (http://bmjopen.bmj.com).

If you have any questions on BMJ Open's open peer review process please email info.bmjopen@bmj.com

BMJ Open

The efficacy of artemisinin-based and quinine-based treatments for uncomplicated falciparum malaria in pregnancy: a protocol for systematic review and individual patient data (IPD) meta-analysis

Journal:	*BMJ Open*
Manuscript ID	bmjopen-2018-027503
Article Type:	Protocol
Date Submitted by the Author:	25-Oct-2018

Complete List of Authors:	Saito, Makoto; WorldWide Antimalarial Resistance Network (WWARN), ; University of Oxford, Centre for Tropical Disease and Global Health, Nuffield Department of Medicine<br>Mansoor, Rashid; WorldWide Antimalarial Resistance Network (WWARN); University of Oxford, Centre for Tropical Disease and Global Health, Nuffield Department of Medicine<br>Kennon, Kalynn; WorldWide Antimalarial Resistance Network (WWARN); University of Oxford, Centre for Tropical Disease and Global Health, Nuffield Department of Medicine<br>McGready, Rose; Shoklo Malaria Research Unit, Mahidol-Oxford Tropical Medicine Research Unit, Faculty of Tropical Medicine, Mahidol University; Centre for Tropical Medicine and Global Health, Nuffield Department of Medicine, University of Oxford<br>Nosten, François; Centre for Tropical Medicine and Global Health, Nuffield Department of Medicine, University of Oxford; Shoklo Malaria Research Unit, Mahidol-Oxford Tropical Medicine Research Unit, Faculty of Tropical Medicine, Mahidol University<br>Guerin, Philippe; WorldWide Antimalarial Resistance Network (WWARN); University of Oxford, Centre for Tropical Disease and Global Health, Nuffield Department of Medicine<br>Stepniewska, Kasia; WorldWide Antimalarial Resistance Network (WWARN); University of Oxford, Centre for Tropical Disease and Global Health, Nuffield Department of Medicine

Keywords:	malaria, pregnancy, treatment efficacy, individual patient data meta-analysis, artemisinin, quinine

SCHOLARONE™
Manuscripts

The efficacy of artemisinin-based and quinine-based treatments for uncomplicated falciparum malaria in pregnancy: a protocol for systematic review and individual patient data (IPD) meta-analysis

Makoto Satio[1,2,*], Rashid Mansoor[1,2], Kalynn Kennon [1,2], Rose McGready[2,3], François Nosten[2,3], Philippe J Guérin[1,2], Kasia Stepniewska[1,2]

[1]WorldWide Antimalarial Resistance Network (WWARN), Oxford, UK

[2]Centre for Tropical Medicine and Global Health, Nuffield Department of Clinical Medicine, University of Oxford, Oxford, UK

[3]Shoklo Malaria Research Unit (SMRU), Mahidol-Oxford Tropical Medicine Research Unit, Faculty of Tropical Medicine, Mahidol University, Mae Sot, Tak, Thailand

* Corresponding to Makoto Saito

Address: Centre for Tropical Medicine and Global Health, NDM Research Building, Old Road Campus, Roosevelt Drive, Oxford, OX3 7FZ, UK

Tel: +44-01865 612900

E-mails: makoto.saito@wwarn.org (MS), rashid.mansoor@wwarn.org (RM), kalynn.kennon@wwarn.org (KK), rose@shoklo-unit.com (RoM), francois@tropmedres.ac (FN), philippe.guerin@wwarn.org (PG), kasia.stepniewska@wwarn.org (KS)

Word count: 2957

Key words: malaria, pregnancy, treatment efficacy, individual patient data meta-analysis, artemisinin, quinine

For peer review only

Abstract

Introduction

Pregnant women are more vulnerable to malaria leading to adverse impact on both mothers and fetuses. However, knowledge on the efficacy and safety of antimalarials in pregnancy is limited by the paucity of randomised control trials and the lack of standardised protocols in this sub-population. Pooling individual patient data (IPD) for meta-analysis could address in part these limitations to summarise accurately the currently available evidence on treatment efficacy and risk factors of treatment failure.

Methods and analysis

To assess the treatment efficacy of artemisinin-based and quinine-based treatments for uncomplicated falciparum malaria in pregnancy, seven databases (MEDLINE, Embase, Global Health, Cochrane Library, Scopus, Web of Science and LILACS) and two clinical trial registries (ICTRP and ClinicalTrial.gov) were searched. Both interventional and observational cohort studies following up for at least 28 days will be included. IPD of the identified eligible published or unpublished studies will be sought by inviting principal investigators. Raw IPD will be shared through the web-based secure platform developed by WorldWide Antimalarial Resistance Network (WWARN) using the established methodology. The primary objective is to compare the risk of polymerase chain reaction (PCR)-corrected treatment failure among different treatments and to find the risk factors. One-stage IPD meta-analysis by Cox model with shared frailty will be conducted. Sensitivity analyses will assess the effect of studies with different study designs and also those whose IPD are not obtained.

For peer review only

Ethics and Dissemination

This IPD meta-analysis consists of secondary analysis of existing anonymous data and meets the criteria for waiver of ethical review by the Oxford Tropical Research Ethics Committee. The results of this IPD meta-analysis will be disseminated through open-access publications at peer-reviewed journals. The study results will lead to better understanding of malaria treatment in pregnancy, which can be used for clinical decision-making and conducting further studies.

PROSPERO registration number: CRD42018104013.

Article Summary

Strengths and limitations of this study

- The research on the efficacy of antimalarials in pregnancy has been restricted by the paucity of randomised control trials and the lack of a standard study design for pregnancy.
- This will be the first individual patient data (IPD) meta-analysis on the efficacy of currently recommended antimalarials in pregnancy incorporating IPD from both interventional and observational cohort studies.
- IPD that are standardised in the same format and analysed in a uniform way with adjustment of covariates, will allow us to compare the efficacy of different treatment as well as to find risk factors of treatment failure in this vulnerable but neglected population.
- The collection of IPD globally will ensure the generalisability of the results.

For peer review only

- Limitations of this IPD meta-analysis include the potential difficulty in acquiring the IPD and the heterogeneity of the study designs, study population and parasite population. A risk of bias analysis will be conducted to address the impact of potential unshared data.

For peer review only

Introduction

About 60% of all pregnancies are estimated to take place in malaria endemic areas.[1] In addition, pregnant women are amongst the most vulnerable groups for malaria infection leading to higher morbidity and mortality of both mothers and fetuses.[2] Although around 1500 studies on the efficacy of antimalarials in malaria treatment have been conducted, pregnant women have been excluded from the majority of clinical trials in the past, mainly because of safety concerns for the fetus.

Due to the lack of evidence for both efficacy and safety, quinine, rather than artemisinin-based combination therapy (ACT), has been recommended as the first-line treatment of uncomplicated *Plasmodium falciparum* malaria for pregnant women in the first trimester by the World Health Organization (WHO).[3] However, recent studies measuring the safety of artemisinin derivatives during pregnancy, including in the first trimester, have shown reassuring results[4-7] and it is likely that ACT will be recommended as the first-line treatment option for pregnant women regardless of the trimester in the next WHO guideline.[8] Nevertheless, the bulk of evidence is limited.

The efficacy and safety of antimalarials in pregnancy can be different from the results from the non-pregnant populations because of altered immunity, physiological change in pharmacokinetics and sequestration of parasites to placenta. Besides, the risk factors of treatment failure in pregnancy need to be assessed to improve the clinical care in pregnancy. However, there are no agreed guidelines on how to assess the efficacy in pregnancy while it is standardised in the non-pregnant patients by WHO.[9] This lack of standard methodology makes it challenging to conduct efficacy studies in pregnancy and leads to the variability of assessing and reporting the

For peer review only

outcomes.[10,11] Taken together, the current situation limits conducting aggregated data meta-analyses.[11]

The WorldWide Antimalarial Resistance Network (WWARN) has established a unique individual participant data (IPD)-sharing platform facilitating large-scale pooled meta-analyses. We plan to include both published and unpublished studies exploring the efficacy and safety of the treatment of malaria during pregnancy. We will conduct a one-stage IPD meta-analysis on the currently recommended antimalarial drugs, i.e. artemisinin-based and quinine-based treatments, used for the treatment of uncomplicated falciparum malaria in pregnancy.

Objectives

The aim of this study is to evaluate and compare treatment outcomes of artemisinin-based and quinine-based treatment for uncomplicated falciparum malaria in pregnancy.

Primary objectives are:

- To compare antimalarial efficacies among artemisinin-based and quinine-based treatments
- To identify risk factors associated with treatment failure
- To assess the relationship between the dosing (dose per body weight) of artemisinin-based treatments and treatment efficacy

Secondary objectives are:

- To evaluate the risk of gametocyte carriage following antimalarial treatment
- To evaluate the risk of adverse events following antimalarial treatment

For peer review only

- To evaluate the risk of *Plasmodium vivax* infection following antimalarial treatment

Methods and analyses

Criteria for study eligibility

Types of studies

- Prospective clinical efficacy studies with a minimum 28-day active follow-up
- Both interventional and observational cohort studies regardless of the number of treatment arms (i.e. comparative or single-arm)
- Genotyping conducted for distinguishing recrudescence and reinfection

The following studies will be excluded.

- ≤ 10 eligible pregnant women
- conducted in non-endemic countries (i.e. returned travellers)

Types of participants

- Pregnant women in any trimester
- Parasitologically confirmed *P. falciparum* parasitaemia
- either asymptomatic or symptomatic

Types of intervention/exposure and controls

- Treated with artemisinin-based or quinine-based treatments

For peer review only

Types of outcomes

- Parasitological and clinical efficacy
- Adverse events

Information sources and search strategy

A systematic literature review was conducted to identify the potential studies to be included in this IPD meta-analysis. Seven databases (MEDLINE, Embase, Global Health, Cochrane Library, Scopus, Web of Science and LILACS) and two clinical trial registries (ICTRP and ClinicalTrial.gov) were used. Both published and unpublished grey literatures such as conference abstracts and registered trials were included. This systematic review and IPD meta-analysis is registered to PROSPERO (CRD42018104013), and the search terms and conditions are available there.

Briefly, the search combined five components: malaria; pregnancy; treatment or names of anti-malarial drugs; study design (interventional or observational cohort studies); and outcome types (efficacy) without limitation on publication year or language. The result of the literature search was published elsewhere.[11] The initial search was conducted on 9 July 2016, and had been updated until 5 July 2018 using PubMed and ICTRP. The IPD meta-analysis will be based on the studies identified by the final updated search.

Data acquisition and data management

Collecting IPD

Principal investigators of the published and unpublished studies identified by the systematic literature review will be invited to share their IPD with WWARN. Emails will be sent

For peer review only

to the corresponding authors on at least three occasions asking whether they are willing to join the study group. A secure web-based platform has been developed by WWARN, and IPD will be uploaded after agreeing to the terms and conditions of the submission.[12] Data are fully anonymised and handled in compliance with the UK Data Protection Act to protect personal information and patient privacy. Original data are stored on a secure server hosted by the University of Oxford.

Data management

Raw data will be curated in a standardised format using the WWARN Clinical Module data management plan to facilitate pooled IPD meta-analyses.[13] After checking the raw data, any queries on the availability of data, ambiguity of the variables or potential errors will be solved by asking the data contributors. The protocol of the original studies will be sought from the data contributors or from the publication when available. The standardised dataset is made traceable to the raw data by use of a unique person (or episode) ID and study ID. This standardised dataset will be used for the analyses.

Statistical analysis plan

Study populations

Pregnant women will be eligible for the purpose of this analysis if they meet the following criteria:

- Confirmed pregnancy status on day 0 of the treatment
- Information on the type, date and dose of antimalarial drugs: artemisinin-based and quinine-based treatments will be included
- Baseline data on patient age and estimated gestational age (or trimester of pregnancy)

For peer review only

- Date of the last day of follow-up or length of follow-up

The following patients will be excluded:

- No or missing data on parasitological confirmation of *P. falciparum* infection at enrolment
- Presenting with severe malaria symptoms at enrolment as defined by WHO[3], except uncomplicated hyperparasitaemia
- Incomplete dose

Outcomes

The primary outcome will be the polymerase chain reaction (PCR)-corrected *P. falciparum* recrudescence. Secondary outcomes will include PCR-corrected *P. falciparum* reinfection; PCR-uncorrected *P. falciparum* recurrence; parasite clearance; gametocyte carriage during follow up; *P. vivax* infection during follow-up; and adverse events that developed after drug administration

Recurrences of *P. falciparum* will be distinguished by PCR into recrudescence (treatment failure) and reinfection.[14] Indeterminate PCR will be regarded as being censored on the day of recurrence in survival analyses for PCR-corrected outcomes. In studies where peripheral malaria smears were examined regularly (e.g. every week), the time of parasite recurrence will be defined as the time of the first positive parasite smear after the parasite clearance following the treatment. For pregnant women with no recurrent parasitaemia recorded, the day of their last negative smear will be regarded as their last visit and censoring time. In case of intermittent follow-up (e.g. missed follow-ups), the following rules will be applied:

(i) Blood smears will be assumed negative between the two negative observations

For peer review only

(ii) If patient came back to be followed up with a positive smear, the date of positive parasitaemia will be assumed to be the date of observation if this date is within 31 days from the last observation

(iii) If parasite clearance is not recorded after treatment but the positive parasite count is recorded at least 7 days after starting the treatment, day of the first positive count will be treated as day of recurrence

Definitions of status and other censorship are detailed in the Clinical Module DMSAP[13] except the above modification. Early late treatment failure is not applied for quinine-based treatment because quinine is given for 7 days.

Adverse symptoms will include: abdominal pain, dizziness, headache, body pain/myalgia, weakness/fatigue, vomiting, nausea, anorexia and tinnitus if data permit.

Variables and their definitions

The following baseline characteristics of patients will be included as appropriate if enough data will be shared: age; estimated gestational age (or trimester); parity or gravidity; weight (weight before pregnancy and weight at treatment); body mass index (BMI); baseline parasitaemia; presence of fever (body temperature > 37.5 degrees Celsius); haemoglobin (or haematocrit); anaemia (Hb < 11 g/dL or Hct < 30% for anaemia and Hb < 7 g/dL or Hct < 20% for severe anaemia);[15] gametocytes on presentation; past history of malaria (time from the last treatment or the number of past infection in the same pregnancy); description of infection (mixed species infections); total mg/kg dose for each drug component; and supervision of drug administration. The doses of drugs received will be calculated from the number of tablets administered to each

For peer review only

For peer review only - http://bmjopen.bmj.com/site/about/guidelines.xhtml

patient. If the actual number of tablets received was not recorded, doses according to the protocol will be used.

For each study, study locations and local transmission intensity will be considered. The study sites will be classified into three categories: low, medium and high malaria transmission based on the parasite prevalence estimates obtained from the Malaria Atlas Project for specific location and year of study.[16,17]

P. vivax intercalated infection will be regarded as censored if the original study did not test PCR for falciparum recurrences after intercalated vivax infection. If the original study tested PCR for falciparum recurrences regardless of intercalated vivax infection, vivax infection will be regarded as a time-dependent covariate.

Descriptive summaries

A summary of the studies and baseline characteristics of the patients included in the analysis will be presented. The number of available patients will be summarised for all variables listed above, proportion will be used for categorical or binary variables, and mean and standard deviation (or median and range) will be used for continuous variables.

PCR-corrected and uncorrected outcomes will be used to compute the Kaplan-Meier (K-M) estimates. The K-M estimates will be presented graphically together with the number of pregnant women in the risk set. Log rank test will be used for assessing the overall K-M profiles between treatment groups. The efficacy between treatments will be compared at fixed time points (i.e. on day 28, 42, and 63) by constructing a chi-squared test statistics using the stratified (by study sites) approach.[18]

For peer review only

231 **Analysis of primary outcome**

232 A one-stage IPD meta-analysis using Cox model with shared frailty will be conducted to
233 identify the risk factors of treatment failure as well as comparing different treatments. Study sites
234 will be fitted as random effects. For repeated episodes, multi-level mixed effects model (if there
235 are enough data) or the previous history of malaria will be used. If data permit, non-linear
236 relationship will be examined for continuous variables.[19] Cox-Snell and Schoenfeld residuals will
237 be examined to determine the appropriateness of model fit and proportional hazard assumption,
238 respectively. Alternative statistical approaches such as flexible parametric models or introducing
239 an interaction with time will be considered if the proportionality assumption is not satisfied.

240 **Analyses of secondary outcomes**

241 Analysis of secondary outcomes will be carried out provided enough data is present; else,
242 only summary statistics will be reported. Analyses similar to the primary outcome will be
243 conducted for *P. falciparum* reinfection, *P. falciparum* recurrence and *P. vivax* infection.

244 Parasite clearance will be assessed as the proportions of patients cleared asexual falciparum
245 parasitaemia on day 1, 2 and 3. Univariable and multivariable mixed effect logistic regression
246 models (or Cox models for the time to parasite clearance) will be used to identify the risk factors
247 associated with parasite positivity status.

248 Gametocyte carriage will be assessed as the proportion of patients with *P. falciparum*
249 gametocytes on day 0, 3, 7, 14 or 21. Proportions after day 0 will be stratified by the presence of
250 gametocytes at baseline. If enough data is available, mixed effects logistic regression models will
251 be used to assess the risk factors for gametocytes carriage on day 0 and after treatment stratified
252 by the presence of gametocytes at baseline.

For peer review only

Adverse symptoms will be assessed as the proportion of patients who developed adverse symptoms after the treatment initiation. Proportions of patients who developed symptoms after day 0 will be stratified by whether or not that symptom was present before the treatment initiation. If enough data are available, mixed effects logistic regression models will be used to assess the risk factors of adverse symptoms developed after the treatment initiation. Symptoms on day 0 (before treatment) will be added as a covariate. Primarily the symptoms developed in the first week will be included. Symptoms developed at any time during the study period may be added. Pregnancy outcomes may be assessed if enough data will be gathered.

For peer review only

Variable selection

For any regression models, the following strategy recommended by Collet [20] will be used to determine independent risk factors. Initially all possible risk factors will be examined in the univariable model to assess if any of the variables are related to the treatment outcome. All significant variables with a p-value ≤ 0.05 will then be added to the baseline model. The variables with p-value of > 0.05 will be excluded from the baseline model one by one starting from the variable with the largest p-value. Once only significant covariates remained in the model, all excluded variables will be added to this model one by one to check whether there are any variables that become significant in the presence of other risk factors. Likelihood Ratio Test (LRT) and Akaike's Information Criterion (AIC) will be used to compare nested and non-nested models, respectively. Treatment and baseline parasitaemia will be included in the multivariable models as *a priori* forced variables regardless of the statistical significance. Variables that are missing more than 50% will not be included in multivariable analyses.[21] Interaction between gravidity (parity) and endemicity will be assessed, as the impact of gravidity (i.e. pregnancy-specific immunity) can be different depending on the endemicity.

Assessment of statistical heterogeneity across studies

The multilevel logistic or Cox models would be used for explaining the study-site heterogeneity. Heterogeneity across study sites will be statistically assessed as the variance of the shared frailty term estimated in the random effect Cox model or variance of the random intercepts in logistic regression. Additionally, intra-class correlation in logistic regression model will be reported.

Subgroup analyses

Analyses will be conducted by geographical regions and by treatment (for assessing dose impact of each drug) if data permit.

Sensitivity analyses

Three types of sensitivity analyses will be performed. Firstly, a model will be refitted excluding non-RCTs to assess whether the baseline imbalance due to observational cohort studies or single-arm interventional studies will influence the results. Secondly, a model will be refitted with excluding one study at a time to identify any influential studies. Thirdly, to assess the impact of covariates with missing values, multiple imputation may be used.[21]

Strength of the body of evidence / risk of bias across studies

The risk of bias within and across the included studies will be assessed following the GRADE guideline.[22] Publication bias will be evaluated by a funnel plot of the log-transformed hazards ratio (odds ratio or proportion),[23] if more than 10 studies will be included.[24] Despite the effort, all the studies identified in the systematic review may not be shared and included in this IPD meta-analysis. The bias by the studies that are unable to be included in the analyses will be evaluated as a sensitivity analysis.[25] The reported aggregated efficacy will be extracted from the publication and a two-stage meta-analysis combining shared and unshared data will be attempted.[26]

For peer review only - http://bmjopen.bmj.com/site/about/guidelines.xhtml

Further development of statistical analysis plan

The main analysis is planned as described above. Modification or additional analyses may be required as the data collection progresses. Updated statistical analysis plans will be available at the WWARN website.[27]

Software

Statistical analysis will be conducted using R (The R Foundation for Statistical Computing) or STATA (College Station, Texas, USA).

Ethics and dissemination

This IPD meta-analysis met the criteria for waiver of ethical review as defined by the Oxford Tropical Research Ethics Committee (OxTREC) as the research consists of secondary analysis of existing anonymous data.[28] All studies included in this analysis will have received local ethical approvals and our pooled IPD meta-analysis will be addressing scientific questions that are very similar to the original research questions.

Findings will be reported following the PRISMA-IPD guideline[29] at peer-reviewed journals with open access. The progress will be updated on our study group website.[27] This protocol is reported following PRISMA-P guideline[30,31] and the systematic literature review and IPD meta-analysis is registered to PROSPERO (CRD42018104013). Any publications based on the findings of this IPD meta-analysis will be in accordance with the guidelines of the International Committee of Medical Journal Editors.

For peer review only

Discussion

This IPD meta-analysis will update the previous aggregated data meta-analyses that included only four or five RCTs,[11,32] by incorporating the IPD from single-arm interventional or observational cohort studies. As the data can be standardised and analysed in a uniform way, IPD meta-analyses are particularly useful when there is no standard study design such as in this case. Risk factors associated with treatment failures particularly the dosing of the currently used treatments can be assessed in IPD meta-analyses, but rarely in aggregated data meta-analyses. Although meta-analyses of secondary data cannot include variables that were not assessed in the original studies, the results of this IPD meta-analysis can identify the pregnant women in need of close clinical monitoring based on what is commonly assessed. Despite the increased time and effort of gathering and standardising the IPD, the advantages of IPD meta-analysis outweigh particularly for answering research questions on these neglected minority populations.

WWARN has developed the secure and equitable data platform and the international collaborative network of malaria researchers worldwide over the last decade. With this unique collaborative effort, we hope that these findings will lead to the improvement of clinical management of this vulnerable but neglected population.

Author Contributions

MS, RoM and PG conceived the idea. MS and RM drafted the manuscript. KK, FN, RoM, PG and KS critically revised the manuscript. All authors have read and approved the final manuscript.

For peer review only

Funding

The WorldWide Antimalarial Resistance Network (WWARN) is funded by Bill and Melinda Gates Foundation. SMRU is part of the Mahidol Oxford University Research Unit supported by the Wellcome Trust of Great Britain. MS is supported by the University of Oxford Clarendon Fund. The funders did not participate in the study development, the writing of the paper, decision to publish, or preparation of the manuscript.

Competing interests

None declared.

For peer review only

Ethics approval

This individual patient data meta-analysis met the criteria for waiver of ethical review as defined by the Oxford Tropical Research Ethics Committee (OxTREC) since the research consists of secondary analysis of existing anonymous data. Each study included in the analysis will have received local ethics approvals.

Acknowledgement

We would like to thank Dr Mary Ellen Gilder for her contribution to the previous systematic literature review.

References

1. Dellicour S, Tatem AJ, Guerra CA, et al. Quantifying the number of pregnancies at risk of malaria in 2007: a demographic study. *PLoS Med* 2010;7(1):e1000221.

2. Rogerson SJ, Desai M, Mayor A, et al. Burden, pathology, and costs of malaria in pregnancy: new developments for an old problem. *Lancet Infect Dis* 2018;18(4):e107-e18.
3. WHO. Guidelines for the treatment of malaria. Third edition. Geneva: World Health Organization 2015.
4. Dellicour S, Sevene E, McGready R, et al. First-trimester artemisinin derivatives and quinine treatments and the risk of adverse pregnancy outcomes in Africa and Asia: A meta-analysis of observational studies. *PLoS Med* 2017;14(5):e1002290.
5. Kovacs SD, van Eijk AM, Sevene E, et al. The Safety of Artemisinin Derivatives for the Treatment of Malaria in the 2nd or 3rd Trimester of Pregnancy: A Systematic Review and Meta-Analysis. *PLoS One* 2016;11(11):e0164963.
6. McGready R, Lee SJ, Wiladphaingern J, et al. Adverse effects of falciparum and vivax malaria and the safety of antimalarial treatment in early pregnancy: a population-based study. *Lancet Infect Dis* 2012;12(5):388-96.
7. Moore KA, Simpson JA, Paw MK, et al. Safety of artemisinins in first trimester of prospectively followed pregnancies: an observational study. *Lancet Infect Dis* 2016;16(5):576-83.
8. WHO Malaria Policy Advisory Committee Secretariat. Malaria Policy Advisory Committee to the WHO: conclusions and recommendations of eighth biannual meeting (September 2015). *Malar J* 2016;15:117.
9. World Health Organization. Methods for surveillance of antimalarial drug efficacy. Geneva: World Health Organization 2009.
10. Saito M, Gilder ME, Nosten F, et al. Methodology of assessment and reporting of safety in anti-malarial treatment efficacy studies of uncomplicated falciparum malaria in pregnancy: a systematic literature review. *Malar J* 2017;16:491.
11. Saito M, Gilder ME, Nosten F, et al. Systematic literature review and meta-analysis of the efficacy of artemisinin-based and quinine-based treatments for uncomplicated falciparum malaria in pregnancy: methodological challenges. *Malar J* 2017;16:488.
12. WorldWide Antimalarial Resistance Network (WWARN). Terms of Submission 2017 [cited 5 July 2018]. Available from: http://www.wwarn.org/tools-resources/terms-submission.
13. WorldWide Antimalarial Resistance Network (WWARN). Clinical Module: Data Management and Statistical Analysis Plan Version 1.2. 2012 [cited 5 July 2018]. Available from: http://www.wwarn.org/sites/default/files/ClinicalDMSAP.pdf.
14. World Health Organization. Methods and techniques for clinical trials on antimalarial drug efficacy: Genotyping to identify parasite populations. Geneva: World Health Organization 2008.
15. World Health Organization. Haemoglobin concentrations for the diagnosis of anaemia and assessment of severity. Geneva: World Health Organization 2011.
16. Bhatt S, Weiss DJ, Cameron E, et al. The effect of malaria control on Plasmodium falciparum in Africa between 2000 and 2015. *Nature* 2015;526(7572):207-11.
17. Gething PW, Patil AP, Smith DL, et al. A new world malaria map: Plasmodium falciparum endemicity in 2010. *Malar J* 2011;10:378.
18. Klein JP, Logan B, Harhoff M, et al. Analyzing survival curves at a fixed point in time. *Stat Med* 2007;26(24):4505-19.
19. Royston P, Sauerbrei W. Fractional Polynomials for One Variable. Multivariable Model-building: A pragmatic approach to regression analysis based on fractional polynomials for modelling continuous variables. Chuchester: John Wiley & Sons 2008:71-98.

For peer review only

20. Collett D. 3.6 Strategy for model selection. Modelling survival data in medical research Third edition. New York: CRC Press 2015:83-90.
21. White IR, Royston P, Wood AM. Multiple imputation using chained equations: Issues and guidance for practice. *Stat Med* 2011;30(4):377-99.
22. Guyatt G, Oxman AD, Akl EA, et al. GRADE guidelines: 1. Introduction—GRADE evidence profiles and summary of findings tables. *J Clin Epidemiol* 2011;64(4):383-94.
23. Silcocks P. Hazard ratio funnel plots for survival comparisons. *J Epidemiol Community Health* 2009;63(10):856-61.
24. Sterne JA, Sutton AJ, Ioannidis JP, et al. Recommendations for examining and interpreting funnel plot asymmetry in meta-analyses of randomised controlled trials. *BMJ* 2011;343:d4002.
25. Ahmed I, Sutton AJ, Riley RD. Assessment of publication bias, selection bias, and unavailable data in meta-analyses using individual participant data: a database survey. *BMJ* 2012;344
26. Riley RD, Lambert PC, Abo-Zaid G. Meta-analysis of individual participant data: rationale, conduct, and reporting. *BMJ* 2010;340:c221.
27. WorldWide Antimalarial Resistance Network (WWARN). Malaria in pregnancy treatment efficacy study group 2016 [cited 5 July 2018]. Available from: http://www.wwarn.org/working-together/study-groups/malaria-pregnancy-treatment-efficacy-study-group.
28. Oxford Tropical Research Ethics Committee. 2018 [cited 23 October 2018]. Available from: https://researchsupport.admin.ox.ac.uk/governance/ethics/committees/oxtrec.
29. Stewart LA, Clarke M, Rovers M, et al. Preferred Reporting Items for Systematic Review and Meta-Analyses of individual participant data: the PRISMA-IPD Statement. *JAMA* 2015;313(16):1657-65.
30. Shamseer L, Moher D, Clarke M, et al. Preferred reporting items for systematic review and meta-analysis protocols (PRISMA-P) 2015: elaboration and explanation. *BMJ* 2015;349
31. Moher D, Shamseer L, Clarke M, et al. Preferred reporting items for systematic review and meta-analysis protocols (PRISMA-P) 2015 statement. *Systematic Reviews* 2015;4(1):1.
32. Burger RJ, van Eijk AM, Bussink M, et al. Artemisinin-based combination therapy versus quinine or other combinations for treatment of uncomplicated Plasmodium falciparum malaria in the second and third trimester of pregnancy: a systematic review and meta-analysis. *Open Forum Infect Dis* 2016;3(1):ofv170.

For peer review only

PRISMA-P 2015 Checklist

This checklist has been adapted for use with protocol submissions to *Systematic Reviews* from Table 3 in Moher D et al: Preferred reporting items for systematic review and meta-analysis protocols (PRISMA-P) 2015 statement. *Systematic Reviews* 2015 **4**:1

Section/topic	#	Checklist item	Information reported		Line number(s)
			Yes	No	
**ADMINISTRATIVE INFORMATION**					
**Title**					
Identification	1a	Identify the report as a protocol of a systematic review	✓	☐	2-3
Update	1b	If the protocol is for an update of a previous systematic review, identify as such	☐	☐	NA
**Registration**	2	If registered, provide the name of the registry (e.g., PROSPERO) and registration number in the Abstract	✓	☐	53
**Authors**					
Contact	3a	Provide name, institutional affiliation, and e-mail address of all protocol authors; provide physical mailing address of corresponding author	✓	☐	5-20
Contributions	3b	Describe contributions of protocol authors and identify the guarantor of the review	✓	☐	346-349
**Amendments**	4	If the protocol represents an amendment of a previously completed or published protocol, identify as such and list changes; otherwise, state plan for documenting important protocol amendments	☐	☐	NA
**Support**					
Sources	5a	Indicate sources of financial or other support for the review	✓	☐	350-355
Sponsor	5b	Provide name for the review funder and/or sponsor	✓	☐	350-355
Role of sponsor/funder	5c	Describe roles of funder(s), sponsor(s), and/or institution(s), if any, in developing the protocol	✓	☐	350-355
**INTRODUCTION**					
**Rationale**	6	Describe the rationale for the review in the context of what is already known	✓	☐	78-94
**Objectives**	7	Provide an explicit statement of the question(s) the review will address with reference to participants, interventions, comparators, and outcomes (PICO)	✓	☐	95-114

For peer review only

BioMed Central The Open Access Publisher

Section/topic	#	Checklist item	Information reported Yes	Information reported No	Line number(s)
**METHODS**					
**Eligibility criteria**	8	Specify the study characteristics (e.g., PICO, study design, setting, time frame) and report characteristics (e.g., years considered, language, publication status) to be used as criteria for eligibility for the review	✓	☐	116-133
**Information sources**	9	Describe all intended information sources (e.g., electronic databases, contact with study authors, trial registers, or other grey literature sources) with planned dates of coverage	✓	☐	135-141
**Search strategy**	10	Present draft of search strategy to be used for at least one electronic database, including planned limits, such that it could be repeated	✓	☐	135-147
STUDY RECORDS					
Data management	11a	Describe the mechanism(s) that will be used to manage records and data throughout the review	✓	☐	158-165
Selection process	11b	State the process that will be used for selecting studies (e.g., two independent reviewers) through each phase of the review (i.e., screening, eligibility, and inclusion in meta-analysis)	✓	☐	134-157
Data collection process	11c	Describe planned method of extracting data from reports (e.g., piloting forms, done independently, in duplicate), any processes for obtaining and confirming data from investigators	✓	☐	149-165
**Data items**	12	List and define all variables for which data will be sought (e.g., PICO items, funding sources), any pre-planned data assumptions and simplifications	✓	☐	209-228
**Outcomes and prioritization**	13	List and define all outcomes for which data will be sought, including prioritization of main and additional outcomes, with rationale	✓	☐	182-208
**Risk of bias in individual studies**	14	Describe anticipated methods for assessing risk of bias of individual studies, including whether this will be done at the outcome or study level, or both; state how this information will be used in data synthesis	✓	☐	295-309
DATA					
**Synthesis**	15a	Describe criteria under which study data will be quantitatively synthesized	✓	☐	182-228
	15b	If data are appropriate for quantitative synthesis, describe planned summary measures, methods of handling data, and methods of combining data from studies, including any planned exploration of consistency (e.g., I^2, Kendall's tau)	✓	☐	229-291
	15c	Describe any proposed additional analyses (e.g., sensitivity or subgroup analyses, meta-regression)	✓	☐	292-300
	15d	If quantitative synthesis is not appropriate, describe the type of summary planned	✓	☐	307-309

BioMed Central
The Open Access Publisher

Section/topic	#	Checklist item	Information reported		Line number(s)
			Yes	No	
**Meta-bias(es)**	16	Specify any planned assessment of meta-bias(es) (e.g., publication bias across studies, selective reporting within studies)	✓	☐	301-309
**Confidence in cumulative evidence**	17	Describe how the strength of the body of evidence will be assessed (e.g., GRADE)	✓	☐	302-303

For peer review only

BioMed Central The Open Access Publisher

BMJ Open

The efficacy of artemisinin-based and quinine-based treatments for uncomplicated falciparum malaria in pregnancy: a protocol for systematic review and individual patient data (IPD) meta-analysis

Journal:	*BMJ Open*
Manuscript ID	bmjopen-2018-027503.R1
Article Type:	Protocol
Date Submitted by the Author:	31-Mar-2019

Complete List of Authors:	Saito, Makoto; WorldWide Antimalarial Resistance Network (WWARN), ; University of Oxford, Centre for Tropical Disease and Global Health, Nuffield Department of Medicine<br>Mansoor, Rashid; WorldWide Antimalarial Resistance Network (WWARN); University of Oxford, Centre for Tropical Disease and Global Health, Nuffield Department of Medicine<br>Kennon, Kalynn; WorldWide Antimalarial Resistance Network (WWARN); University of Oxford, Centre for Tropical Disease and Global Health, Nuffield Department of Medicine<br>McGready, Rose; Shoklo Malaria Research Unit, Mahidol-Oxford Tropical Medicine Research Unit, Faculty of Tropical Medicine, Mahidol University; Centre for Tropical Medicine and Global Health, Nuffield Department of Medicine, University of Oxford<br>Nosten, François; Centre for Tropical Medicine and Global Health, Nuffield Department of Medicine, University of Oxford; Shoklo Malaria Research Unit, Mahidol-Oxford Tropical Medicine Research Unit, Faculty of Tropical Medicine, Mahidol University<br>Guerin, Philippe; WorldWide Antimalarial Resistance Network (WWARN); University of Oxford, Centre for Tropical Disease and Global Health, Nuffield Department of Medicine<br>Stepniewska, Kasia; WorldWide Antimalarial Resistance Network (WWARN); University of Oxford, Centre for Tropical Disease and Global Health, Nuffield Department of Medicine

**Primary Subject Heading**:	Infectious diseases
Secondary Subject Heading:	Infectious diseases, Pharmacology and therapeutics
Keywords:	malaria, pregnancy, treatment efficacy, individual patient data meta-analysis, artemisinin, quinine

SCHOLARONE™
Manuscripts

The efficacy of artemisinin-based and quinine-based treatments for uncomplicated falciparum malaria in pregnancy: a protocol for systematic review and individual patient data (IPD) meta-analysis

Makoto Saito[1,2,*], Rashid Mansoor[1,2], Kalynn Kennon [1,2], Rose McGready[2,3], François Nosten[2,3], Philippe J Guérin[1,2], Kasia Stepniewska[1,2]

[1]WorldWide Antimalarial Resistance Network (WWARN), Oxford, UK

[2]Centre for Tropical Medicine and Global Health, Nuffield Department of Clinical Medicine, University of Oxford, Oxford, UK

[3]Shoklo Malaria Research Unit (SMRU), Mahidol-Oxford Tropical Medicine Research Unit, Faculty of Tropical Medicine, Mahidol University, Mae Sot, Tak, Thailand

* Corresponding to Makoto Saito

Address: Centre for Tropical Medicine and Global Health, NDM Research Building, Old Road Campus, Roosevelt Drive, Oxford, OX3 7FZ, UK

Tel: +44-01865 612900

E-mails: makoto.saito@wwarn.org (MS), rashid.mansoor@wwarn.org (RM), kalynn.kennon@wwarn.org (KK), rose@shoklo-unit.com (RoM), francois@tropmedres.ac (FN), philippe.guerin@wwarn.org (PG), kasia.stepniewska@wwarn.org (KS)

Word count: 2998

Key words: malaria, pregnancy, treatment efficacy, individual patient data meta-analysis, artemisinin, quinine

For peer review only

Abstract

Introduction

Pregnant women are more vulnerable to malaria leading to adverse impact on both mothers and fetuses. However, knowledge on the efficacy and safety of antimalarials in pregnancy is limited by the paucity of randomised control trials and the lack of standardised protocols in this special sub-population. Pooling individual patient data (IPD) for meta-analysis could address in part these limitations to summarise accurately the currently available evidence on treatment efficacy and risk factors of treatment failure.

Methods and analysis

To assess the treatment efficacy of artemisinin-based and quinine-based treatments for uncomplicated falciparum malaria in pregnancy, seven databases (MEDLINE, Embase, Global Health, Cochrane Library, Scopus, Web of Science and Literatura Latino Americana em Ciências da Saúde) and two clinical trial registries (International Clinical Trials Registry Platform and ClinicalTrial.gov) were searched. Both interventional and observational cohort studies following up for at least 28 days will be included. IPD of the identified eligible published or unpublished studies will be sought by inviting principal investigators. Raw IPD will be shared through the web-based secure platform developed by WorldWide Antimalarial Resistance Network using the established methodology. The primary objective is to compare the risk of polymerase chain reaction (PCR)-corrected treatment failure among different treatments and to find the risk factors. One-stage IPD meta-analysis by Cox model with shared frailty will be conducted. A risk of bias assessment will be conducted to address the impact of potential unshared data and of the quality of individual studies. Potential limitations include difficulty in acquiring the IPD and heterogeneity of the study designs due to the lack of standard.

For peer review only

Ethics and Dissemination

This IPD meta-analysis consists of secondary analyses of existing anonymous data and meets the criteria for waiver of ethical review by the Oxford Tropical Research Ethics Committee. The results of this IPD meta-analysis will be disseminated through open-access publications at peer-reviewed journals. The study results will lead to a better understanding of malaria treatment in pregnancy, which can be used for clinical decision-making and conducting further studies.

PROSPERO registration number: CRD42018104013.

Article Summary

Strengths and limitations of this study

- The research on the efficacy of antimalarials in pregnancy has been restricted by the paucity of randomised control trials and the lack of a standard study design for pregnancy.
- This study will be the first individual patient data (IPD) meta-analysis on the efficacy of currently recommended antimalarials in pregnancy incorporating IPD from both randomised control trials and single-arm cohort studies.
- IPD that are standardised in the same format and analysed in a uniform way with adjustment of covariates will allow us to compare the efficacy of different treatment as well as to find risk factors of treatment failure in this vulnerable but understudied population.
- The collection of IPD globally will ensure the generalisability of the results.

For peer review only

- Limitations of this IPD meta-analysis include the potential difficulty in acquiring the IPD and the heterogeneity of the study designs, study population and parasite population. A risk of bias assessment will be conducted to address the impact of potential unshared data and of the quality of individual studies.

For peer review only

Introduction

About 60% of all pregnancies are estimated to take place in malaria-endemic areas.[1] In addition, pregnant women are amongst the most vulnerable groups for malaria infection leading to higher morbidity and mortality of both mothers and fetuses.[2] Although around 1500 studies on the efficacy of antimalarials in malaria treatment have been conducted,[3] pregnant women have been excluded from the majority of clinical trials in the past, mainly because of safety concerns for the fetus.

Due to the lack of evidence for both efficacy and safety of antimalarials in pregnancy, quinine, rather than artemisinin-based combination therapy (ACT), has been recommended as the first-line treatment of uncomplicated *Plasmodium falciparum* malaria for pregnant women in the first trimester by the World Health Organization (WHO).[4] However, recent studies measuring the safety of artemisinin derivatives during pregnancy, including in the first trimester, have shown reassuring results[5-8] and it is likely that ACT will be recommended as the first-line treatment option for pregnant women regardless of the trimester in the next WHO guidelines.[9] Evidence on the treatment efficacy during pregnancy needs to be assembled.

The efficacy and safety of antimalarials in pregnancy can be different from the results from the non-pregnant populations because of altered immunity, physiological change in pharmacokinetics and sequestration of parasites to the placenta. The risk factors of treatment failure in pregnancy need to be assessed to improve clinical care in pregnancy. However, there are no agreed guidelines on how to assess the efficacy in pregnancy while it is standardised in the non-pregnant patients by WHO.[10] This lack of standard methodology makes it challenging to conduct efficacy studies in pregnancy and leads to the variability of assessing and reporting the outcomes.[11,12] Taken together, the current situation limits conducting aggregated data meta-analyses.[12]

For peer review only

The WorldWide Antimalarial Resistance Network (WWARN) has established a unique individual participant data (IPD)-sharing platform facilitating large-scale pooled meta-analyses. We plan to include both published and unpublished studies exploring the efficacy and safety of the treatment of malaria during pregnancy. We will conduct a one-stage IPD meta-analysis on the currently recommended antimalarial drugs, i.e. artemisinin-based and quinine-based treatments, used for the treatment of uncomplicated falciparum malaria in pregnancy.

Objectives

The aim of this study is to evaluate and compare treatment outcomes of artemisinin-based and quinine-based treatment for uncomplicated falciparum malaria in pregnancy.

Primary objectives are:

- To compare antimalarial efficacies among artemisinin-based and quinine-based treatments
- To identify risk factors associated with treatment failure

Secondary objectives are:

- To assess the relationship between the dosing (dose per body weight) of artemisinin-based treatments and treatment efficacy
- To evaluate the risk of gametocyte carriage following artemisinin-based and quinine-based treatments
- To evaluate the safety and tolerability of artemisinin-based and quinine-based treatments

For peer review only

117 # Methods and analyses

118 *Criteria for study eligibility*

119 **Types of studies**

120 - Prospective clinical efficacy studies with a minimum 28-day active follow-up
121 - Both interventional and observational cohort studies regardless of the number of
122 treatment arms (i.e. comparative or single-arm)
123 - Genotyping conducted for distinguishing recrudescence and reinfection

124 The following studies will be excluded.

125 - ≤ 10 eligible pregnant women
126 - Conducted in non-endemic countries (i.e. returned travellers)

127 **Types of participants**

128 - Pregnant women in any trimester
129 - Parasitologically confirmed *P. falciparum* parasitaemia
130 - Either asymptomatic or symptomatic

131 **Types of intervention/exposure and controls**

132 - Treated with artemisinin-based or quinine-based treatments

133 **Types of outcomes**

134 - Parasitological and clinical efficacy
135 - Adverse events

For peer review only

Information sources and search strategy

A systematic literature review was conducted to identify the potential studies to be included in this IPD meta-analysis. Seven databases (MEDLINE, Embase, Global Health, Cochrane Library, Scopus, Web of Science and Literatura Latino Americana em Ciências da Saúde) and two clinical trial registries (International Clinical Trials Registry Platform and ClinicalTrial.gov) were used. Both published and unpublished grey literature such as conference abstracts and registered trials were included. This systematic review and IPD meta-analysis is registered to PROSPERO (CRD42018104013), and the search terms and conditions are available there.

Briefly, the search combined five components: malaria; pregnancy; treatment or names of anti-malarial drugs; study design (interventional or observational cohort studies); and outcome types (efficacy) without limitation on publication year or language. The result of the literature search was published elsewhere.[12] The initial search was conducted on 9 July 2016. The final search will be updated in April 2019.

Data acquisition and data management

Collecting IPD

Principal investigators of the published and unpublished studies identified by the systematic literature review will be invited to share their IPD with WWARN. Emails will be sent to the corresponding authors on at least three occasions asking whether they are willing to join the study group. A secure web-based platform has been developed by WWARN, and IPD will be uploaded after agreeing to the terms and conditions of the submission, retaining the ownership and full control of their shared data.[13] Data are fully anonymised and handled in compliance with the UK Data Protection Act to protect personal information and patient privacy. Original data are stored on a secure server hosted by the University of Oxford.

For peer review only

Data management

Raw data will be curated in a standardised format using the WWARN Clinical Module data management plan to facilitate pooled IPD meta-analyses.[14] After checking the raw data, any queries on the availability of data, ambiguity of the variables or potential errors will be resolved by asking the data contributors. The protocol of the original studies will be sought from the data contributors or the publication when available. The standardised dataset will be used for the analyses.

Statistical analysis plan

Study populations

Pregnant women will be eligible for the purpose of this analysis if they meet the following criteria:

- Confirmed pregnancy status on day 0 of the treatment
- Information on the type, date and dose of antimalarial drugs: artemisinin-based and quinine-based treatments will be included
- Baseline data on patient age and estimated gestational age (or trimester of pregnancy)
- Date of the last day of follow-up or length of follow-up

The following patients will be excluded:

- No or missing data on parasitological confirmation of *P. falciparum* infection at enrolment
- Presenting with severe malaria symptoms at enrolment as defined by WHO[4], except uncomplicated hyperparasitaemia

For peer review only

Outcomes

The primary outcome will be the polymerase chain reaction (PCR)-corrected *P. falciparum* treatment failure. Secondary outcomes will include any recurrence of malaria (PCR-uncorrected treatment failure); parasite clearance; gametocyte carriage during follow up; and adverse events that developed after drug administration.

Recurrences of *P. falciparum* will be distinguished by PCR into recrudescence (treatment failure) and reinfection.[15] Indeterminate PCR will be excluded, and reinfection will be regarded as being censored on the day of recurrence in survival analyses for PCR-corrected outcomes following the WHO guidelines.[10] In studies where peripheral malaria smears were examined regularly (e.g. every week), the time of parasite recurrence will be defined as the time of the first positive parasite smear after the parasite clearance following the treatment. For pregnant women with no recurrent parasitaemia recorded, the day of their last negative smear will be regarded as their last visit and censoring time. In the case of intermittent follow-up (e.g. missed follow-ups), the following rules will be applied:

(i) Blood smears will be assumed negative between the two negative observations

(ii) If a patient came back to be followed up with a positive smear, the date of positive parasitaemia will be assumed to be the date of observation if this date is within 28 (±3) days from the last observation

(iii) If parasite clearance is not recorded after treatment but the positive parasite count is recorded at least 7 days after starting the treatment, the day of the first positive count will be treated as the day of recurrence

Definitions of status and other censorship are detailed in the Clinical Module DMSAP[14] except for the above modification. The presence of parasitaemia within the first seven days will

For peer review only

not be regarded as treatment failure for quinine-based treatment because quinine is given for seven days.

Adverse symptoms will include: abdominal pain, dizziness, headache, body pain/myalgia, weakness/fatigue, vomiting, nausea, anorexia and tinnitus if data permit.

Variables and their definitions

The following baseline characteristics of patients will be included as appropriate if enough data are shared: age; estimated gestational age (or trimester); parity or gravidity; weight (weight before pregnancy and weight at treatment); body mass index (BMI); baseline parasitaemia; presence of fever (body temperature > 37.5 degrees Celsius); haemoglobin (or haematocrit); anaemia (Hb < 11 g/dL or Hct < 30% for anaemia and Hb < 7 g/dL or Hct < 20% for severe anaemia);[16] gametocytes on presentation; past history of malaria or antimalaria use description of infection (mixed species infections); total mg/kg dose for each drug component; and supervision of drug administration. The doses of drugs received will be calculated from the number of tablets administered to each patient. If the actual number of tablets received was not recorded, doses according to the protocol will be used. Only those who completed the standard dose will be included in the primary analysis. The proportion of partial treatment will be presented.

For peer review only

For each study, study locations and local transmission intensity will be considered. The study sites will be classified into three categories: low, medium and high malaria transmission based on the parasite prevalence estimates obtained from the Malaria Atlas Project for specific location and year of study.[17,18]

Plasmodium vivax intercalated infection (i.e. *P. vivax* mono-infection before the recurrence of *P. falciparum* parasitaemia) will be regarded as censored if the original study did not test PCR for falciparum recurrences after intercalated vivax infection, following the WHO

guidelines.[10] If the original study tested PCR for falciparum recurrences regardless of intercalated vivax infection, vivax infection will be regarded as a time-dependent covariate.

Descriptive summaries

A summary of the studies and baseline characteristics of the patients included in the analysis will be presented. The number of available patients will be summarised for all variables listed above, proportion will be used for categorical or binary variables, and mean and standard deviation (or median and interquartile range) will be used for continuous variables.

PCR-corrected and uncorrected outcomes will be used to compute the Kaplan-Meier (K-M) estimates. The efficacy of each treatment will be summarised at fixed time points (i.e. on day 28, 42, and 63) by constructing a chi-squared test statistics using the stratified (by study sites) approach.[19]

Analysis of primary outcome

A one-stage IPD meta-analysis using the Cox model with shared frailty will be conducted to identify the risk factors of treatment failure as well as comparing different treatments. Study sites will be fitted as random effects. For repeated episodes, if any, multi-level mixed effects model (if there are enough data) or the previous history of malaria will be used. If data permit, a non-linear relationship will be examined for continuous variables.[20] Cox-Snell and Schoenfeld residuals will be examined to determine the appropriateness of model fit and proportional hazard assumption, respectively. Alternative statistical approaches such as flexible parametric models or introducing an interaction term with time will be considered if the proportionality assumption is not satisfied.

For peer review only

Analyses of secondary outcomes

Analysis of secondary outcomes will be carried out provided enough data are present; else, only summary statistics will be reported. Analyses similar to the primary outcome will be conducted for PCR-uncorrected treatment failure (i.e. any recurrence of malaria).

Parasite clearance will be assessed as the proportions of patients cleared asexual falciparum parasitaemia on day 1, 2 and 3. Univariable and multivariable mixed-effects logistic regression models (or Cox models for the time to parasite clearance) will be used to identify the risk factors associated with parasite positivity status.

Gametocyte carriage will be assessed as the proportion of patients with *P. falciparum* gametocytes on day 0, 3, 7, 14 or 21. Proportions after day 0 will be stratified by the presence of gametocytes at baseline. If enough data are available, mixed effects logistic regression models will be used to assess the risk factors for gametocytes carriage after treatment stratified by the presence of gametocytes at baseline.

Adverse effects will be assessed as the proportion of patients who developed symptoms after the treatment initiation. Proportions of patients who developed symptoms after day 0 will be stratified by whether or not that symptom was present before the treatment initiation. If enough data are available, mixed effects logistic regression models will be used to assess the risk factors of adverse symptoms developed after the treatment initiation. Symptoms on day 0 (before treatment) will be added as a covariate. Primarily the symptoms developed in the first week will be included. Pregnancy outcomes and placental malaria may be assessed if enough data are gathered.

Variable selection

For any regression models, the following strategy recommended by Collet [21] will be used to determine independent risk factors. Initially, all possible risk factors will be examined

For peer review only

in the univariable model to assess if any of the variables are related to the treatment outcome. All significant variables with a p-value ≤ 0.05 will then be added to the baseline model. The variables with a p-value of > 0.05 will be excluded from the baseline model one by one starting from the variable with the largest p-value. Once only significant covariates will remain in the model, all excluded variables will be added to this model one by one to check whether there will be any variables that become significant in the presence of other risk factors. Likelihood Ratio Test (LRT) and Akaike's Information Criterion (AIC) will be used to compare nested and non-nested models, respectively. Treatment and baseline parasitaemia will be included in the multivariable models on treatment efficacy as *a priori* forced variables regardless of the statistical significance. Variables that are missing more than 50% will not be included in multivariable analyses.[22] Interaction between gravidity (parity) and endemicity, or age and endemicity will be assessed if age or gravidity is included in the multivariable model, as the impact of age and gravidity (i.e. pregnancy-specific immunity) can be different depending on the endemicity.[23]

Assessment of statistical heterogeneity across studies

The multilevel logistic or Cox models would be used for explaining the study-site heterogeneity. Heterogeneity across study sites will be statistically assessed as the variance of the shared frailty term estimated in the random effect Cox model or variance of the random intercepts in logistic regression. Additionally, the intra-class correlation in logistic regression model will be reported.

Subgroup analyses

Analyses will be conducted by malaria transmission intensity and by treatment (for assessing dose impact of each drug) if data permit.

For peer review only

For peer review only - http://bmjopen.bmj.com/site/about/guidelines.xhtml

Sensitivity analyses

Two types of sensitivity analyses will be performed. Firstly, a model will be refitted with excluding one study at a time to identify any influential studies. Secondly, to assess the impact of covariates with missing values, multiple imputation may be used.[22]

Strength of the body of evidence / risk of bias across studies

The risk of bias within and across the included studies will be assessed following the GRADE guidelines.[24] Publication bias will be evaluated by a funnel plot of the log-transformed hazards ratio (odds ratio or proportion),[25] if more than ten studies are included.[26] Despite the effort, all the studies identified in the systematic review may not be shared and included in this IPD meta-analysis. The bias by the studies that are unable to be included in the analyses will be evaluated.[27] The reported aggregated efficacy will be extracted from the publication and compared to the studies included. A two-stage meta-analysis combining shared and unshared data will be attempted if data permit.[28] The impact of artemisinin resistance in the study year at the study site will be evaluated by using the reported prevalence on molecular resistance marker (K-13).

Further development of statistical analysis plan

The main analysis is planned as described above. Modification or additional analyses may be required as the data collection progresses. Updated statistical analysis plans will be available at the WWARN website if an amendment is required.[29]

Software

Statistical analysis will be conducted using R (The R Foundation for Statistical Computing) or Stata MP 14.2 (College Station, Texas, USA).

For peer review only

Patient and Public Involvement

This IPD meta-analysis will use existing secondary data. Patients and public were not involved in the design, recruitment or conduct of this IPD meta-analysis. The results of this study will be shared with the primary investigators of the shared studies and disseminated as publications in open access journals.

Ethics and dissemination

This IPD meta-analysis met the criteria for waiver of ethical review as defined by the Oxford Tropical Research Ethics Committee (OxTREC) as the research consists of secondary analyses of existing anonymous data.[30] All studies included in this analysis will have received local ethical approvals.

Findings will be reported following the PRISMA-IPD guideline[31] at peer-reviewed journals with open access. The progress will be updated on our study group website.[29] This protocol is reported following PRISMA-P guideline[32,33] and the systematic literature review and IPD meta-analysis is registered to PROSPERO (CRD42018104013). Any publications based on the findings of this IPD meta-analysis will be in accordance with the guidelines of the International Committee of Medical Journal Editors.

Discussion

This IPD meta-analysis will update the previous aggregated data meta-analyses that included only four or five RCTs.[12,34] In IPD meta-analyses, data from single-arm interventional or observational cohort studies can be included. As the data can be standardised and analysed in a uniform way, IPD meta-analyses are particularly useful when there is no standard study design such as in this case. Risk factors associated with treatment failures particularly the dosing of the currently used treatments can be assessed in IPD meta-analyses, but rarely in

For peer review only

aggregated data meta-analyses. Although meta-analyses of secondary data cannot include variables that were not assessed in the original studies, the results of this IPD meta-analysis can identify the pregnant women in need of close clinical monitoring based on what is commonly assessed. Despite the increased time and effort of gathering and standardising the IPD, the advantages of IPD meta-analysis outweigh particularly for answering research questions on these neglected or understudied populations.

WWARN has developed the secure and equitable data platform and the international collaborative network of malaria researchers worldwide over the last decade. With this unique collaborative effort, we hope that these findings will lead to the improvement of clinical management of this vulnerable but understudied population.

Author Contributions

MS, RoM and PG conceived the idea. MS and RM drafted the manuscript. KK, FN, RoM, PG and KS critically revised the manuscript. All authors have read and approved the final manuscript.

Funding

The WorldWide Antimalarial Resistance Network (WWARN) is funded by Bill and Melinda Gates Foundation. SMRU is part of the Mahidol Oxford University Research Unit supported by the Wellcome Trust of Great Britain. MS is supported by the University of Oxford Clarendon Fund. The funders did not participate in the study development, the writing of the paper, decision to publish, or preparation of the manuscript.

For peer review only

Competing interests

None declared.

Ethics approval

This individual patient data meta-analysis met the criteria for waiver of ethical review as defined by the Oxford Tropical Research Ethics Committee (OxTREC) since the research consists of secondary analyses of existing anonymous data. Each study included in the analysis will have received local ethics approvals.

Acknowledgement

We would like to thank Dr Mary Ellen Gilder for her contribution to the previous systematic literature review and Dr Prabin Dahal for his comments on the draft.

References

1. Dellicour S, Tatem AJ, Guerra CA, et al. Quantifying the number of pregnancies at risk of malaria in 2007: a demographic study. *PLoS Med* 2010;7(1):e1000221.
2. Rogerson SJ, Desai M, Mayor A, et al. Burden, pathology, and costs of malaria in pregnancy: new developments for an old problem. *Lancet Infect Dis* 2018;18(4):e107-e18.
3. WorldWide Antimalarial Resistance Network. WWARN clinical trials publication library 2017 [cited 20 March 2019] Available from: https://www.wwarn.org/tools-resources/literature-reviews/wwarn-clinical-trials-publication-library.
4. World Health Organization. Guidelines for the treatment of malaria. Third edition. Geneva: World Health Organization 2015.
5. Dellicour S, Sevene E, McGready R, et al. First-trimester artemisinin derivatives and quinine treatments and the risk of adverse pregnancy outcomes in Africa and Asia: A meta-analysis of observational studies. *PLoS Med* 2017;14(5):e1002290.
6. Kovacs SD, van Eijk AM, Sevene E, et al. The Safety of Artemisinin Derivatives for the Treatment of Malaria in the 2nd or 3rd Trimester of Pregnancy: A Systematic Review and Meta-Analysis. *PLoS One* 2016;11(11):e0164963.
7. McGready R, Lee SJ, Wiladphaingern J, et al. Adverse effects of falciparum and vivax malaria and the safety of antimalarial treatment in early pregnancy: a population-based study. *Lancet Infect Dis* 2012;12(5):388-96.

For peer review only

8. Moore KA, Simpson JA, Paw MK, et al. Safety of artemisinins in first trimester of prospectively followed pregnancies: an observational study. *Lancet Infect Dis* 2016;16(5):576-83.

9. WHO Malaria Policy Advisory Committee Secretariat. Malaria Policy Advisory Committee to the WHO: conclusions and recommendations of eighth biannual meeting (September 2015). *Malar J* 2016;15:117.

10. World Health Organization. Methods for surveillance of antimalarial drug efficacy. Geneva: World Health Organization 2009.

11. Saito M, Gilder ME, Nosten F, et al. Methodology of assessment and reporting of safety in anti-malarial treatment efficacy studies of uncomplicated falciparum malaria in pregnancy: a systematic literature review. *Malar J* 2017;16:491.

12. Saito M, Gilder ME, Nosten F, et al. Systematic literature review and meta-analysis of the efficacy of artemisinin-based and quinine-based treatments for uncomplicated falciparum malaria in pregnancy: methodological challenges. *Malar J* 2017;16:488.

13. WorldWide Antimalarial Resistance Network (WWARN). Terms of Submission 2017 [cited 5 July 2018]. Available from: http://www.wwarn.org/tools-resources/terms-submission.

14. WorldWide Antimalarial Resistance Network (WWARN). Clinical Module: Data Management and Statistical Analysis Plan Version 1.2. 2012 [cited 5 July 2018]. Available from: http://www.wwarn.org/sites/default/files/ClinicalDMSAP.pdf.

15. World Health Organization. Methods and techniques for clinical trials on antimalarial drug efficacy: Genotyping to identify parasite populations. Geneva: World Health Organization 2008.

16. World Health Organization. Haemoglobin concentrations for the diagnosis of anaemia and assessment of severity. Geneva: World Health Organization 2011.

17. Bhatt S, Weiss DJ, Cameron E, et al. The effect of malaria control on Plasmodium falciparum in Africa between 2000 and 2015. *Nature* 2015;526(7572):207-11.

18. Gething PW, Patil AP, Smith DL, et al. A new world malaria map: Plasmodium falciparum endemicity in 2010. *Malar J* 2011;10:378.

19. Klein JP, Logan B, Harhoff M, et al. Analyzing survival curves at a fixed point in time. *Stat Med* 2007;26(24):4505-19.

20. Royston P, Sauerbrei W. Fractional Polynomials for One Variable. Multivariable Model-building: A pragmatic approach to regression analysis based on fractional polynomials for modelling continuous variables. Chichester, UK: John Wiley & Sons 2008:71-98.

21. Collett D. 3.6 Strategy for model selection. In: Collett D, ed. Modelling survival data in medical research Third edition. New York: CRC Press 2015:83-90.

22. White IR, Royston P, Wood AM. Multiple imputation using chained equations: Issues and guidance for practice. *Stat Med* 2011;30(4):377-99.

23. Rogerson SJ, Hviid L, Duffy PE, et al. Malaria in pregnancy: pathogenesis and immunity. *Lancet Infect Dis* 2007;7(2):105-17.

24. Guyatt G, Oxman AD, Akl EA, et al. GRADE guidelines: 1. Introduction—GRADE evidence profiles and summary of findings tables. *J Clin Epidemiol* 2011;64(4):383-94.

25. Silcocks P. Hazard ratio funnel plots for survival comparisons. *J Epidemiol Community Health* 2009;63(10):856-61.

26. Sterne JA, Sutton AJ, Ioannidis JP, et al. Recommendations for examining and interpreting funnel plot asymmetry in meta-analyses of randomised controlled trials. *BMJ* 2011;343:d4002.

For peer review only

27. Ahmed I, Sutton AJ, Riley RD. Assessment of publication bias, selection bias, and unavailable data in meta-analyses using individual participant data: a database survey. *BMJ* 2012;344
28. Riley RD, Lambert PC, Abo-Zaid G. Meta-analysis of individual participant data: rationale, conduct, and reporting. *BMJ* 2010;340:c221.
29. WorldWide Antimalarial Resistance Network (WWARN). Malaria in pregnancy treatment efficacy study group 2016 [cited 5 July 2018]. Available from: http://www.wwarn.org/working-together/study-groups/malaria-pregnancy-treatment-efficacy-study-group.
30. Oxford Tropical Research Ethics Committee. 2018 [cited 23 October 2018]. Available from: https://researchsupport.admin.ox.ac.uk/governance/ethics/committees/oxtrec.
31. Stewart LA, Clarke M, Rovers M, et al. Preferred Reporting Items for Systematic Review and Meta-Analyses of individual participant data: the PRISMA-IPD Statement. *JAMA* 2015;313(16):1657-65.
32. Shamseer L, Moher D, Clarke M, et al. Preferred reporting items for systematic review and meta-analysis protocols (PRISMA-P) 2015: elaboration and explanation. *BMJ* 2015;349
33. Moher D, Shamseer L, Clarke M, et al. Preferred reporting items for systematic review and meta-analysis protocols (PRISMA-P) 2015 statement. *Systematic Reviews* 2015;4(1):1.
34. Burger RJ, van Eijk AM, Bussink M, et al. Artemisinin-based combination therapy versus quinine or other combinations for treatment of uncomplicated Plasmodium falciparum malaria in the second and third trimester of pregnancy: a systematic review and meta-analysis. *Open Forum Infect Dis* 2016;3(1):ofv170.

For peer review only

PRISMA-P 2015 Checklist

This checklist has been adapted for use with protocol submissions to *Systematic Reviews* from Table 3 in Moher D et al: Preferred reporting items for systematic review and meta-analysis protocols (PRISMA-P) 2015 statement. *Systematic Reviews* 2015 **4**:1

Section/topic	#	Checklist item	Information reported		Line number(s)
			Yes	No	
**ADMINISTRATIVE INFORMATION**					
**Title**					
Identification	1a	Identify the report as a protocol of a systematic review	✓	☐	2-3
Update	1b	If the protocol is for an update of a previous systematic review, identify as such	☐	☐	NA
**Registration**	2	If registered, provide the name of the registry (e.g., PROSPERO) and registration number in the Abstract	✓	☐	53
**Authors**					
Contact	3a	Provide name, institutional affiliation, and e-mail address of all protocol authors; provide physical mailing address of corresponding author	✓	☐	5-20
Contributions	3b	Describe contributions of protocol authors and identify the guarantor of the review	✓	☐	346-349
**Amendments**	4	If the protocol represents an amendment of a previously completed or published protocol, identify as such and list changes; otherwise, state plan for documenting important protocol amendments	☐	☐	NA
**Support**					
Sources	5a	Indicate sources of financial or other support for the review	✓	☐	350-355
Sponsor	5b	Provide name for the review funder and/or sponsor	✓	☐	350-355
Role of sponsor/funder	5c	Describe roles of funder(s), sponsor(s), and/or institution(s), if any, in developing the protocol	✓	☐	350-355
**INTRODUCTION**					
**Rationale**	6	Describe the rationale for the review in the context of what is already known	✓	☐	78-94
**Objectives**	7	Provide an explicit statement of the question(s) the review will address with reference to participants, interventions, comparators, and outcomes (PICO)	✓	☐	95-114

For peer review only

Section/topic	#	Checklist item	Information reported		Line number(s)
			Yes	No	
**METHODS**					
**Eligibility criteria**	8	Specify the study characteristics (e.g., PICO, study design, setting, time frame) and report characteristics (e.g., years considered, language, publication status) to be used as criteria for eligibility for the review	✓	☐	116-133
**Information sources**	9	Describe all intended information sources (e.g., electronic databases, contact with study authors, trial registers, or other grey literature sources) with planned dates of coverage	✓	☐	135-141
**Search strategy**	10	Present draft of search strategy to be used for at least one electronic database, including planned limits, such that it could be repeated	✓	☐	135-147
STUDY RECORDS					
Data management	11a	Describe the mechanism(s) that will be used to manage records and data throughout the review	✓	☐	158-165
Selection process	11b	State the process that will be used for selecting studies (e.g., two independent reviewers) through each phase of the review (i.e., screening, eligibility, and inclusion in meta-analysis)	✓	☐	134-157
Data collection process	11c	Describe planned method of extracting data from reports (e.g., piloting forms, done independently, in duplicate), any processes for obtaining and confirming data from investigators	✓	☐	149-165
**Data items**	12	List and define all variables for which data will be sought (e.g., PICO items, funding sources), any pre-planned data assumptions and simplifications	✓	☐	209-228
**Outcomes and prioritization**	13	List and define all outcomes for which data will be sought, including prioritization of main and additional outcomes, with rationale	✓	☐	182-208
**Risk of bias in individual studies**	14	Describe anticipated methods for assessing risk of bias of individual studies, including whether this will be done at the outcome or study level, or both; state how this information will be used in data synthesis	✓	☐	295-309
DATA					
**Synthesis**	15a	Describe criteria under which study data will be quantitatively synthesized	✓	☐	182-228
	15b	If data are appropriate for quantitative synthesis, describe planned summary measures, methods of handling data, and methods of combining data from studies, including any planned exploration of consistency (e.g., I^2, Kendall's tau)	✓	☐	229-291
	15c	Describe any proposed additional analyses (e.g., sensitivity or subgroup analyses, meta-regression)	✓	☐	292-300
	15d	If quantitative synthesis is not appropriate, describe the type of summary planned	✓	☐	307-309

For peer review only

BioMed Central The Open Access Publisher

Section/topic	#	Checklist item	Information reported Yes	Information reported No	Line number(s)
**Meta-bias(es)**	16	Specify any planned assessment of meta-bias(es) (e.g., publication bias across studies, selective reporting within studies)	✓	☐	301-309
**Confidence in cumulative evidence**	17	Describe how the strength of the body of evidence will be assessed (e.g., GRADE)	✓	☐	302-303

For peer review only

BioMed Central The Open Access Publisher

BMJ Open

The efficacy of artemisinin-based and quinine-based treatments for uncomplicated falciparum malaria in pregnancy: a protocol for systematic review and individual patient data (IPD) meta-analysis

Journal:	*BMJ Open*
Manuscript ID	bmjopen-2018-027503.R2
Article Type:	Protocol
Date Submitted by the Author:	26-Jun-2019
Complete List of Authors:	Saito, Makoto; WorldWide Antimalarial Resistance Network (WWARN), ; University of Oxford, Centre for Tropical Disease and Global Health, Nuffield Department of Medicine<br>Mansoor, Rashid; WorldWide Antimalarial Resistance Network (WWARN); University of Oxford, Centre for Tropical Disease and Global Health, Nuffield Department of Medicine<br>Kennon, Kalynn; WorldWide Antimalarial Resistance Network (WWARN); University of Oxford, Centre for Tropical Disease and Global Health, Nuffield Department of Medicine<br>McGready, Rose; Shoklo Malaria Research Unit, Mahidol-Oxford Tropical Medicine Research Unit, Faculty of Tropical Medicine, Mahidol University; Centre for Tropical Medicine and Global Health, Nuffield Department of Medicine, University of Oxford<br>Nosten, François; Centre for Tropical Medicine and Global Health, Nuffield Department of Medicine, University of Oxford; Shoklo Malaria Research Unit, Mahidol-Oxford Tropical Medicine Research Unit, Faculty of Tropical Medicine, Mahidol University<br>Guerin, Philippe; WorldWide Antimalarial Resistance Network (WWARN); University of Oxford, Centre for Tropical Disease and Global Health, Nuffield Department of Medicine<br>Stepniewska, Kasia; WorldWide Antimalarial Resistance Network (WWARN); University of Oxford, Centre for Tropical Disease and Global Health, Nuffield Department of Medicine
**Primary Subject Heading**:	Infectious diseases
Secondary Subject Heading:	Infectious diseases, Pharmacology and therapeutics, Obstetrics and gynaecology
Keywords:	malaria, pregnancy, treatment efficacy, individual patient data meta-analysis, artemisinin, quinine

The efficacy of artemisinin-based and quinine-based treatments for uncomplicated falciparum malaria in pregnancy: a protocol for systematic review and individual patient data (IPD) meta-analysis

Makoto Saito[1,2,*], Rashid Mansoor[1,2], Kalynn Kennon [1,2], Rose McGready[2,3], François Nosten[2,3], Philippe J Guérin[1,2], Kasia Stepniewska[1,2]

[1]WorldWide Antimalarial Resistance Network (WWARN), Oxford, UK

[2]Centre for Tropical Medicine and Global Health, Nuffield Department of Clinical Medicine, University of Oxford, Oxford, UK

[3]Shoklo Malaria Research Unit (SMRU), Mahidol-Oxford Tropical Medicine Research Unit, Faculty of Tropical Medicine, Mahidol University, Mae Sot, Tak, Thailand

* Corresponding to Makoto Saito

Address: Centre for Tropical Medicine and Global Health, NDM Research Building, Old Road Campus, Roosevelt Drive, Oxford, OX3 7FZ, UK

Tel: +44-01865 612900

E-mails: makoto.saito@wwarn.org (MS), rashid.mansoor@wwarn.org (RM), kalynn.kennon@wwarn.org (KK), rose@shoklo-unit.com (RoM), francois@tropmedres.ac (FN), philippe.guerin@wwarn.org (PG), kasia.stepniewska@wwarn.org (KS)

Word count: 2998

Key words: malaria, pregnancy, treatment efficacy, individual patient data meta-analysis, artemisinin, quinine

For peer review only

Abstract

Introduction

Pregnant women are more vulnerable to malaria leading to adverse impact on both mothers and fetuses. However, knowledge on the efficacy and safety of antimalarials in pregnancy is limited by the paucity of randomised control trials and the lack of standardised protocols in this special sub-population. Pooling individual patient data (IPD) for meta-analysis could address in part these limitations to summarise accurately the currently available evidence on treatment efficacy and risk factors for treatment failure.

Methods and analysis

To assess the treatment efficacy of artemisinin-based and quinine-based treatments for uncomplicated falciparum malaria in pregnancy, seven databases (MEDLINE, Embase, Global Health, Cochrane Library, Scopus, Web of Science and Literatura Latino Americana em Ciências da Saúde) and two clinical trial registries (International Clinical Trials Registry Platform and ClinicalTrial.gov) were searched. Both interventional and observational cohort studies following up for at least 28 days will be included. IPD of the identified eligible published or unpublished studies will be sought by inviting principal investigators. Raw IPD will be shared through the web-based secure platform developed by WorldWide Antimalarial Resistance Network using the established methodology. The primary objective is to compare the risk of polymerase chain reaction (PCR)-corrected treatment failure among different treatments and to find the risk factors. One-stage IPD meta-analysis by Cox model with shared frailty will be conducted. A risk of bias assessment will be conducted to address the impact of potential unshared data and of the quality of individual studies. Potential limitations include difficulty in acquiring the IPD and heterogeneity of the study designs due to the lack of standard.

For peer review only

Ethics and Dissemination

This IPD meta-analysis consists of secondary analyses of existing anonymous data and meets the criteria for waiver of ethics review by the Oxford Tropical Research Ethics Committee. The results of this IPD meta-analysis will be disseminated through open-access publications at peer-reviewed journals. The study results will lead to a better understanding of malaria treatment in pregnancy, which can be used for clinical decision-making and conducting further studies.

PROSPERO registration number: CRD42018104013.

Article Summary

Strengths and limitations of this study

- This study will be the first individual patient data (IPD) meta-analysis on the efficacy of currently recommended antimalarials in pregnancy incorporating IPD from both randomised control trials and single-arm cohort studies, overcoming the limitation of aggregated data meta-analysis that can only include randomised control trials.
- IPD that are standardised in the same format and analysed in a uniform way with adjustment of covariates will, in contrast to aggregated data, allow us to compare the efficacy of different treatments as well as to find risk factors for treatment failure in this vulnerable but understudied population.
- Limitations of this IPD meta-analysis include the potential difficulty in acquiring the IPD and the heterogeneity of the study designs, study population and parasite population. A risk of bias assessment will be conducted to address the impact of potential unshared data and of the quality of individual studies.

For peer review only

Introduction

About 60% of all pregnancies are estimated to take place in malaria-endemic areas.[1] In addition, pregnant women are amongst the most vulnerable groups for malaria infection leading to higher morbidity and mortality of both mothers and fetuses.[2] Although around 1500 studies on the efficacy of antimalarials in malaria treatment have been conducted,[3] pregnant women have been excluded from the majority of clinical trials in the past, mainly because of safety concerns for the fetus.

Due to the lack of evidence for both efficacy and safety of antimalarials in pregnancy, quinine, rather than artemisinin-based combination therapy (ACT), has been recommended as the first-line treatment of uncomplicated *Plasmodium falciparum* malaria for pregnant women in the first trimester by the World Health Organization (WHO).[4] However, recent studies measuring the safety of artemisinin derivatives during pregnancy, including in the first trimester, have shown reassuring results[5-8] and it is likely that ACT will be recommended as the first-line treatment option for pregnant women regardless of the trimester in the next WHO treatment guidelines.[9] Evidence on the treatment efficacy during pregnancy needs to be assembled.

The efficacy and safety of antimalarials in pregnancy can be different from the results from the non-pregnant populations because of altered immunity, physiological change in pharmacokinetics and sequestration of parasites to the placenta. The risk factors for treatment failure in pregnancy need to be assessed to improve clinical care in pregnancy. However, there are no agreed guidelines on how to assess the efficacy in pregnancy while it is standardised in the non-pregnant patients by WHO.[10] This lack of standard methodology makes it challenging to conduct efficacy studies in pregnancy and leads to the variability of assessing and reporting

For peer review only

the outcomes.[11 12] Taken together, the current situation limits conducting aggregated data meta-analyses.[12]

The WorldWide Antimalarial Resistance Network (WWARN) has established a unique individual participant data (IPD)-sharing platform facilitating large-scale pooled meta-analyses. We plan to include both published and unpublished studies exploring the efficacy and safety of the treatment of malaria during pregnancy. We will conduct a one-stage IPD meta-analysis on the currently recommended antimalarial drugs, i.e. artemisinin-based and quinine-based treatments, used for the treatment of uncomplicated falciparum malaria in pregnancy.

Objectives

The aim of this study is to evaluate and compare treatment outcomes of artemisinin-based and quinine-based treatment for uncomplicated falciparum malaria in pregnancy.

Primary objectives are:

- To compare antimalarial efficacies among artemisinin-based and quinine-based treatments
- To identify risk factors associated with treatment failure

Secondary objectives are:

- To assess the relationship between the dosing (dose per body weight) of artemisinin-based treatments and treatment efficacy
- To evaluate the risk of gametocyte carriage following artemisinin-based and quinine-based treatments
- To evaluate the safety and tolerability of artemisinin-based and quinine-based treatments

For peer review only

Methods and analyses

Criteria for study eligibility

Types of studies

- Prospective clinical efficacy studies with a minimum 28-day active follow-up
- Both interventional and observational cohort studies regardless of the number of treatment arms (i.e. comparative or single arm)
- Genotyping conducted for distinguishing recrudescence and reinfection

The following studies will be excluded.

- ≤ 10 eligible pregnant women
- Conducted in non-endemic countries (i.e. returned travellers)

Types of participants

- Pregnant women in any trimester
- Parasitologically confirmed *P. falciparum* parasitaemia
- Either asymptomatic or symptomatic

Types of intervention/exposure and controls

- Treated with artemisinin-based or quinine-based treatments

Types of outcomes

- Parasitological and clinical efficacy
- Adverse events

For peer review only

Information sources and search strategy

A systematic literature review was conducted to identify the potential studies to be included in this IPD meta-analysis. Seven databases (MEDLINE, Embase, Global Health, Cochrane Library, Scopus, Web of Science and Literatura Latino Americana em Ciências da Saúde) and two clinical trial registries (International Clinical Trials Registry Platform and ClinicalTrial.gov) were used. Both published and unpublished grey literature such as conference abstracts and registered trials were included. This systematic review and IPD meta-analysis is registered to PROSPERO (CRD42018104013), and the search terms and conditions are available there.

Briefly, the search combined five components: malaria; pregnancy; treatment or names of anti-malarial drugs; study design (interventional or observational cohort studies); and outcome types (efficacy) without limitation on publication year or language. The result of the literature search was published elsewhere.[12] The initial search was conducted on 9 July 2016. The final search will be updated in April 2019.

Data acquisition and data management

Collecting IPD

Principal investigators of the published and unpublished studies identified by the systematic literature review will be invited to share their IPD with WWARN. Emails will be sent to the corresponding authors on at least three occasions asking whether they are willing to join the study group. A secure web-based platform has been developed by WWARN, and IPD will be uploaded after agreeing to the terms and conditions of the submission, retaining the ownership and full control of their shared data.[13] Data are fully anonymised and handled in compliance with the UK Data Protection Act to protect personal information and patient privacy. Original data are stored on a secure server hosted by the University of Oxford.

For peer review only

Data management

Raw data will be curated in a standardised format using the WWARN Clinical Module data management plan to facilitate pooled IPD meta-analyses.[14] After checking the raw data, any queries on the availability of data, ambiguity of the variables or potential errors will be resolved by asking the data contributors. The protocol of the original studies will be sought from the data contributors or the publication when available. The standardised dataset will be used for the analyses.

Statistical analysis plan

Study populations

Pregnant women will be eligible for the purpose of this analysis if they meet the following criteria:

- Confirmed pregnancy status on day 0 of the treatment
- Information on the type, date and dose of antimalarial drugs: artemisinin-based and quinine-based treatments will be included
- Baseline data on patient age and estimated gestational age (or trimester of pregnancy)
- Date of the last day of follow-up or length of follow-up

The following patients will be excluded:

- No or missing data on parasitological confirmation of *P. falciparum* infection at enrolment
- Presenting with severe malaria symptoms at enrolment as defined by WHO[4], except hyperparasitaemia and severe anaemia, which will be included

For peer review only

Outcomes

The primary outcome will be the polymerase chain reaction (PCR)-corrected *P. falciparum* treatment failure. Secondary outcomes will include any recurrence of malaria (PCR-uncorrected treatment failure); parasite clearance; gametocyte carriage during follow up; and adverse events that developed after drug administration.

Recurrences of *P. falciparum* will be distinguished by PCR into recrudescence (treatment failure) and reinfection.[15] Indeterminate PCR will be excluded, and reinfection will be regarded as being censored on the day of recurrence in survival analyses for PCR-corrected outcomes following the WHO guidelines.[10] In studies where peripheral malaria smears were examined regularly (e.g. every week), the time of parasite recurrence will be defined as the time of the first positive parasite smear after the parasite clearance following the treatment. For pregnant women with no recurrent parasitaemia recorded, the day of their last negative smear will be regarded as their last visit and censoring time. In the case of intermittent follow-up (e.g. missed follow-ups), the following rules will be applied:

(i) Blood smears will be assumed negative between the two negative observations

(ii) If a patient came back to be followed up with a positive smear, the date of positive parasitaemia will be assumed to be the date of observation if this date is within 28 (±3) days from the last observation

(iii) If parasite clearance is not recorded after treatment but the positive parasite count is recorded at least 7 days after starting the treatment, the day of the first positive count will be treated as the day of recurrence

Definitions of status and other censorship are detailed in the Clinical Module data management plan [14] except for the above modification. The presence of parasitaemia within

For peer review only

the first seven days will not be regarded as treatment failure for quinine-based treatment because quinine is given for seven days.

Adverse symptoms will include abdominal pain, dizziness, headache, body pain/myalgia, weakness/fatigue, vomiting, nausea, anorexia and tinnitus if data permit.

Variables and their definitions

The following baseline characteristics of patients will be included as appropriate if enough data are shared: age; estimated gestational age (or trimester); parity or gravidity; weight (weight before pregnancy and weight at treatment); body mass index (BMI); baseline parasitaemia; presence of fever (body temperature > 37.5 degrees Celsius); haemoglobin (or haematocrit); anaemia (haemoglobin < 11 g/dL or haematocrit < 30% for anaemia and haemoglobin < 7 g/dL or haematocrit < 20% for severe anaemia);[16] gametocytes on presentation; past history of malaria or antimalaria use; description of infection (mixed species infections); total mg/kg dose for each drug component; and supervision of drug administration. The doses of drugs received will be calculated from the number of tablets administered to each patient. If the actual number of tablets received was not recorded, doses according to the protocol will be used. Only those who completed the standard dose will be included in the primary analysis. The proportion of partial treatment will be presented.

For each study, study locations and local transmission intensity will be considered. The study sites will be classified into three categories: low, medium and high malaria transmission based on the parasite prevalence estimates obtained from the Malaria Atlas Project for specific location and year of study.[17,18]

Plasmodium vivax intercalated infection (i.e. *P. vivax* mono-infection before the recurrence of *P. falciparum* parasitaemia) will be regarded as censored if the original study did not test PCR for falciparum recurrences after intercalated vivax infection, following the WHO

For peer review only

guidelines.[10] If the original study tested PCR for falciparum recurrences regardless of intercalated vivax infection, vivax infection will be regarded as a time-dependent covariate.

Descriptive summaries

A summary of the studies and baseline characteristics of the patients included in the analysis will be presented. The number of available patients will be summarised for all variables listed above, proportion will be used for categorical or binary variables, and mean and standard deviation (or median and interquartile range) will be used for continuous variables.

PCR-corrected and uncorrected outcomes will be used to compute the Kaplan-Meier (K-M) estimates for each study site. The efficacy of each treatment will then be summarised at fixed time points (i.e. on day 28, 42, and 63) by the aggregated meta-analysis approach.

Analysis of primary outcome

A one-stage IPD meta-analysis using the Cox model with shared frailty for study sites will be conducted to identify the risk factors for treatment failure as well as comparing different treatments. For repeated episodes, if any, multi-level mixed-effects model (if there are enough data) or the previous history of malaria will be used. If data permit, a non-linear relationship will be examined for continuous variables.[19] Cox-Snell and Schoenfeld residuals will be examined to determine the appropriateness of model fit and proportional hazard assumption, respectively. Alternative statistical approaches such as flexible parametric models or introducing an interaction term with time will be considered if the proportionality assumption is not satisfied.

Analyses of secondary outcomes

Analysis of secondary outcomes will be carried out provided enough data are present; else, only summary statistics will be reported. Analyses similar to the primary outcome will be conducted for PCR-uncorrected treatment failure (i.e. any recurrence of malaria).

For peer review only

Parasite clearance will be assessed as the proportions of patients cleared asexual falciparum parasitaemia on day 1, 2 and 3. Univariable and multivariable mixed-effects logistic regression models (or Cox models for the time to parasite clearance) will be used to identify the risk factors associated with parasite positivity status.

Gametocyte carriage will be assessed as the proportion of patients with *P. falciparum* gametocytes on day 0, 3, 7, 14 or 21. Proportions after day 0 will be stratified by the presence of gametocytes at baseline. If enough data are available, mixed-effects logistic regression models will be used to assess the risk factors for gametocytes carriage after treatment stratified by the presence of gametocytes at baseline.

Adverse effects will be assessed as the proportion of patients who developed symptoms after the treatment initiation. Proportions of patients who developed symptoms after day 0 will be stratified by whether or not that symptom was present before the treatment initiation. If enough data are available, mixed-effects logistic regression models will be used to assess the risk factors for adverse symptoms developed after the treatment initiation. Symptoms on day 0 (before treatment) will be added as a covariate. Primarily the symptoms developed in the first week will be included. Pregnancy outcomes and placental malaria may be assessed if enough data are gathered.

Variable selection

For any regression models, the following strategy recommended by Collet [20] will be used to determine independent risk factors. Initially, all possible risk factors will be examined in the univariable model to assess if any of the variables are related to the treatment outcome. All significant variables with a p-value ≤ 0.05 will then be added to the baseline model. The variables with a p-value of > 0.05 will be excluded from the baseline model one by one starting from the variable with the largest p-value. Once only significant covariates will remain in the

For peer review only

model, all excluded variables will be added to this model one by one to check whether there will be any variables that become significant in the presence of other risk factors. Likelihood Ratio Test (LRT) and Akaike's Information Criterion (AIC) will be used to compare nested and non-nested models, respectively. Treatment and baseline parasitaemia will be included in the multivariable models on treatment efficacy as *a priori* forced variables regardless of the statistical significance. Variables that are missing more than 50% will not be included in multivariable analyses.[21] Interaction between gravidity (parity) and endemicity, or age and endemicity will be assessed if age or gravidity is included in the multivariable model, as the impact of age and gravidity (i.e. pregnancy-specific immunity) can be different depending on the endemicity.[22]

Assessment of statistical heterogeneity across studies

The multilevel logistic or Cox models would be used for explaining the study-site heterogeneity. Heterogeneity across study sites will be statistically assessed as the variance of the shared frailty term estimated in the Cox model or variance of the random intercepts in logistic regression. Additionally, the intra-class correlation in logistic regression model will be reported.

Subgroup analyses

Analyses will be conducted by malaria transmission intensity and by treatment (for assessing dose impact of each drug) if data permit.

Sensitivity analyses

Two types of sensitivity analyses will be performed. Firstly, a model will be refitted with excluding one study at a time to identify any influential studies. Secondly, to assess the impact of covariates with missing values, multiple imputation may be used.[21]

For peer review only

Strength of the body of evidence / risk of bias across studies

The risk of bias within and across the included studies will be assessed following the GRADE guidelines.[23] Publication bias will be evaluated by a funnel plot of the log-transformed hazards ratio (odds ratio or proportion),[24] if more than ten studies are included.[25] Despite the effort, all the studies identified in the systematic review may not be shared and included in this IPD meta-analysis. The bias by the studies that are unable to be included in the analyses will be evaluated.[26] The reported aggregated efficacy will be extracted from the publication and compared with the studies included. A two-stage meta-analysis combining shared and unshared data will be attempted if data permit.[27] The impact of artemisinin resistance in the study year at the study site will be evaluated by using the reported prevalence on molecular resistance marker (K-13).

Further development of statistical analysis plan

The main analysis is planned as described above. Modification or additional analyses may be required as the data collection progresses. Updated statistical analysis plans will be available at the WWARN website if an amendment is required.[28]

Software

Statistical analysis will be conducted using R (The R Foundation for Statistical Computing, Vienna, Austria) or Stata MP 15.1 (StataCorp, College Station, Texas, USA).

Patient and Public Involvement

This IPD meta-analysis will use existing secondary data. Patients and public were not involved in the design, recruitment or conduct of this IPD meta-analysis. The results of this study will be shared with the primary investigators of the shared studies and disseminated as publications in open access journals.

For peer review only

Ethics and dissemination

This IPD meta-analysis met the criteria for waiver of ethics review as defined by the Oxford Tropical Research Ethics Committee (OxTREC) as the research consists of secondary analyses of existing anonymous data.[29] All studies included in this analysis will have received local ethical approvals.

Findings will be reported following the PRISMA-IPD guideline[30] at peer-reviewed journals with open access. The progress will be updated on our study group website.[28] This protocol is reported following PRISMA-P guideline[31 32] and the systematic literature review and IPD meta-analysis is registered to PROSPERO (CRD42018104013). Any publications based on the findings of this IPD meta-analysis will be in accordance with the guidelines of the International Committee of Medical Journal Editors.

Discussion

This IPD meta-analysis will update the previous aggregated data meta-analyses that included only four or five RCTs.[12 33] In IPD meta-analyses, data from single-arm interventional or observational cohort studies can be included. As the data can be standardised and analysed in a uniform way, IPD meta-analyses are particularly useful when there is no standard study design such as in this case. Risk factors associated with treatment failures particularly the dosing of the currently used treatments can be assessed in IPD meta-analyses, but rarely in aggregated data meta-analyses. Although meta-analyses of secondary data cannot include variables that were not assessed in the original studies, the results of this IPD meta-analysis can identify the pregnant women in need of close clinical monitoring based on what is commonly assessed. Despite the increased time and effort of gathering and standardising the

For peer review only

IPD, the advantages of IPD meta-analysis outweigh particularly for answering research questions on these neglected or understudied populations.

WWARN has developed the secure and equitable data platform and the international collaborative network of malaria researchers worldwide over the last decade. With this unique collaborative effort, we hope that these findings will lead to the improvement of clinical management of this vulnerable but understudied population.

Author Contributions

MS, RoM and PG conceived the idea. MS and RM drafted the manuscript. KK, FN, RoM, PG and KS critically revised the manuscript. All authors have read and approved the final manuscript.

Funding

The WorldWide Antimalarial Resistance Network (WWARN) is funded by the Bill and Melinda Gates Foundation and the ExxonMobil Foundation. SMRU is part of the Mahidol Oxford University Research Unit supported by the Wellcome Trust of Great Britain. MS is supported by the University of Oxford Clarendon Fund. The funders did not participate in the study development, the writing of the paper, decision to publish, or preparation of the manuscript.

Competing interests

None declared.

For peer review only

Ethics approval

This individual patient data meta-analysis met the criteria for waiver of ethics review as defined by the Oxford Tropical Research Ethics Committee (OxTREC) since the research consists of secondary analyses of existing anonymous data. Each study included in the analysis will have received local ethics approvals.

Acknowledgement

We would like to thank Dr Mary Ellen Gilder for her contribution to the previous systematic literature review and Dr Prabin Dahal for his comments on the draft.

References

1. Dellicour S, Tatem AJ, Guerra CA, et al. Quantifying the number of pregnancies at risk of malaria in 2007: a demographic study. *PLoS Med* 2010;7(1):e1000221. doi: 10.1371/journal.pmed.1000221
2. Rogerson SJ, Desai M, Mayor A, et al. Burden, pathology, and costs of malaria in pregnancy: new developments for an old problem. *Lancet Infect Dis* 2018;18(4):e107-e18. doi: 10.1016/S1473-3099(18)30066-5
3. WorldWide Antimalarial Resistance Network. WWARN clinical trials publication library 2017 [cited 20 March 2019]. Available from: https://www.wwarn.org/tools-resources/literature-reviews/wwarn-clinical-trials-publication-library.
4. World Health Organization. Guidelines for the treatment of malaria. Third edition. Geneva: WHO Press 2015.
5. Dellicour S, Sevene E, McGready R, et al. First-trimester artemisinin derivatives and quinine treatments and the risk of adverse pregnancy outcomes in Africa and Asia: A meta-analysis of observational studies. *PLoS Med* 2017;14(5):e1002290. doi: 10.1371/journal.pmed.1002290
6. Kovacs SD, van Eijk AM, Sevene E, et al. The Safety of Artemisinin Derivatives for the Treatment of Malaria in the 2nd or 3rd Trimester of Pregnancy: A Systematic Review and Meta-Analysis. *PLoS One* 2016;11(11):e0164963. doi: 10.1371/journal.pone.0164963
7. McGready R, Lee SJ, Wiladphaingern J, et al. Adverse effects of falciparum and vivax malaria and the safety of antimalarial treatment in early pregnancy: a population-based study. *Lancet Infect Dis* 2012;12(5):388-96. doi: 10.1016/S1473-3099(11)70339-5
8. Moore KA, Simpson JA, Paw MK, et al. Safety of artemisinins in first trimester of prospectively followed pregnancies: an observational study. *Lancet Infect Dis* 2016;16(5):576-83. doi: 10.1016/s1473-3099(15)00547-2

For peer review only

9. WHO Malaria Policy Advisory Committee Secretariat. Malaria Policy Advisory Committee to the WHO: conclusions and recommendations of eighth biannual meeting (September 2015). *Malar J* 2016;15:117. doi: 10.1186/s12936-016-1169-x

10. World Health Organization. Methods for surveillance of antimalarial drug efficacy. Geneva: WHO Press 2009.

11. Saito M, Gilder ME, Nosten F, et al. Methodology of assessment and reporting of safety in anti-malarial treatment efficacy studies of uncomplicated falciparum malaria in pregnancy: a systematic literature review. *Malar J* 2017;16:491. doi: 10.1186/s12936-017-2136-x

12. Saito M, Gilder ME, Nosten F, et al. Systematic literature review and meta-analysis of the efficacy of artemisinin-based and quinine-based treatments for uncomplicated falciparum malaria in pregnancy: methodological challenges. *Malar J* 2017;16:488. doi: 10.1186/s12936-017-2135-y

13. WorldWide Antimalarial Resistance Network (WWARN). Terms of Submission 2017 [cited 5 July 2018]. Available from: http://www.wwarn.org/tools-resources/terms-submission.

14. WorldWide Antimalarial Resistance Network (WWARN). Clinical Module: Data Management and Statistical Analysis Plan Version 1.2. 2012 [cited 5 July 2018]. Available from: http://www.wwarn.org/sites/default/files/ClinicalDMSAP.pdf.

15. World Health Organization. Methods and techniques for clinical trials on antimalarial drug efficacy: Genotyping to identify parasite populations. Geneva: WHO Press 2008.

16. World Health Organization. Haemoglobin concentrations for the diagnosis of anaemia and assessment of severity. Geneva: WHO Press 2011.

17. Bhatt S, Weiss DJ, Cameron E, et al. The effect of malaria control on Plasmodium falciparum in Africa between 2000 and 2015. *Nature* 2015;526(7572):207-11. doi: 10.1038/nature15535

18. Gething PW, Patil AP, Smith DL, et al. A new world malaria map: Plasmodium falciparum endemicity in 2010. *Malar J* 2011;10:378. doi: 10.1186/1475-2875-10-378

19. Royston P, Sauerbrei W. Fractional Polynomials for One Variable. Multivariable Model-building: A pragmatic approach to regression analysis based on fractional polynomials for modelling continuous variables. Chichester, UK: John Wiley & Sons 2008:71-98.

20. Collett D. 3.6 Strategy for model selection. Modelling survival data in medical research Third edition. New York: CRC Press 2015:83-90.

21. White IR, Royston P, Wood AM. Multiple imputation using chained equations: Issues and guidance for practice. *Stat Med* 2011;30(4):377-99. doi: 10.1002/sim.4067

22. Rogerson SJ, Hviid L, Duffy PE, et al. Malaria in pregnancy: pathogenesis and immunity. *Lancet Infect Dis* 2007;7(2):105-17. doi: 10.1016/s1473-3099(07)70022-1

23. Guyatt G, Oxman AD, Akl EA, et al. GRADE guidelines: 1. Introduction—GRADE evidence profiles and summary of findings tables. *J Clin Epidemiol* 2011;64(4):383-94. doi: 10.1016/j.jclinepi.2010.04.026

24. Silcocks P. Hazard ratio funnel plots for survival comparisons. *J Epidemiol Community Health* 2009;63(10):856-61. doi: 10.1136/jech.2008.075069

25. Sterne JA, Sutton AJ, Ioannidis JP, et al. Recommendations for examining and interpreting funnel plot asymmetry in meta-analyses of randomised controlled trials. *BMJ* 2011;343:d4002. doi: 10.1136/bmj.d4002

26. Ahmed I, Sutton AJ, Riley RD. Assessment of publication bias, selection bias, and unavailable data in meta-analyses using individual participant data: a database survey. *BMJ* 2012;344 doi: 10.1136/bmj.d7762

27. Riley RD, Lambert PC, Abo-Zaid G. Meta-analysis of individual participant data: rationale, conduct, and reporting. *BMJ* 2010;340:c221. doi: 10.1136/bmj.c221

For peer review only

28. WorldWide Antimalarial Resistance Network (WWARN). Malaria in pregnancy treatment efficacy study group 2016 [cited 5 July 2018]. Available from: http://www.wwarn.org/working-together/study-groups/malaria-pregnancy-treatment-efficacy-study-group.
29. Oxford Tropical Research Ethics Committee. 2018 [cited 23 October 2018]. Available from:https://researchsupport.admin.ox.ac.uk/governance/ethics/committees/oxtrec.
30. Stewart LA, Clarke M, Rovers M, et al. Preferred Reporting Items for Systematic Review and Meta-Analyses of individual participant data: the PRISMA-IPD Statement. *JAMA* 2015;313(16):1657-65. doi: 10.1001/jama.2015.3656
31. Shamseer L, Moher D, Clarke M, et al. Preferred reporting items for systematic review and meta-analysis protocols (PRISMA-P) 2015: elaboration and explanation. *BMJ* 2015;349 doi: 10.1136/bmj.g7647
32. Moher D, Shamseer L, Clarke M, et al. Preferred reporting items for systematic review and meta-analysis protocols (PRISMA-P) 2015 statement. *Systematic Reviews* 2015;4(1):1. doi: 10.1186/2046-4053-4-1
33. Burger RJ, van Eijk AM, Bussink M, et al. Artemisinin-based combination therapy versus quinine or other combinations for treatment of uncomplicated Plasmodium falciparum malaria in the second and third trimester of pregnancy: a systematic review and meta-analysis. *Open Forum Infect Dis* 2016;3(1):ofv170. doi: 10.1093/ofid/ofv170

For peer review only

PRISMA-P 2015 Checklist

This checklist has been adapted for use with protocol submissions to *Systematic Reviews* from Table 3 in Moher D et al: Preferred reporting items for systematic review and meta-analysis protocols (PRISMA-P) 2015 statement. *Systematic Reviews* 2015 **4**:1

Section/topic	#	Checklist item	Information reported		Line number(s)
			Yes	No	
**ADMINISTRATIVE INFORMATION**					
**Title**					
Identification	1a	Identify the report as a protocol of a systematic review	✓	☐	2-3
Update	1b	If the protocol is for an update of a previous systematic review, identify as such	☐	☐	NA
**Registration**	2	If registered, provide the name of the registry (e.g., PROSPERO) and registration number in the Abstract	✓	☐	53
**Authors**					
Contact	3a	Provide name, institutional affiliation, and e-mail address of all protocol authors; provide physical mailing address of corresponding author	✓	☐	5-20
Contributions	3b	Describe contributions of protocol authors and identify the guarantor of the review	✓	☐	346-349
**Amendments**	4	If the protocol represents an amendment of a previously completed or published protocol, identify as such and list changes; otherwise, state plan for documenting important protocol amendments	☐	☐	NA
**Support**					
Sources	5a	Indicate sources of financial or other support for the review	✓	☐	350-355
Sponsor	5b	Provide name for the review funder and/or sponsor	✓	☐	350-355
Role of sponsor/funder	5c	Describe roles of funder(s), sponsor(s), and/or institution(s), if any, in developing the protocol	✓	☐	350-355
**INTRODUCTION**					
**Rationale**	6	Describe the rationale for the review in the context of what is already known	✓	☐	78-94
**Objectives**	7	Provide an explicit statement of the question(s) the review will address with reference to participants, interventions, comparators, and outcomes (PICO)	✓	☐	95-114

BioMed Central The Open Access Publisher

For peer review only - http://bmjopen.bmj.com/site/about/guidelines.xhtml

Section/topic	#	Checklist item	Information reported		Line number(s)
			Yes	No	
**METHODS**					
**Eligibility criteria**	8	Specify the study characteristics (e.g., PICO, study design, setting, time frame) and report characteristics (e.g., years considered, language, publication status) to be used as criteria for eligibility for the review	✓	☐	116-133
**Information sources**	9	Describe all intended information sources (e.g., electronic databases, contact with study authors, trial registers, or other grey literature sources) with planned dates of coverage	✓	☐	135-141
**Search strategy**	10	Present draft of search strategy to be used for at least one electronic database, including planned limits, such that it could be repeated	✓	☐	135-147
STUDY RECORDS					
Data management	11a	Describe the mechanism(s) that will be used to manage records and data throughout the review	✓	☐	158-165
Selection process	11b	State the process that will be used for selecting studies (e.g., two independent reviewers) through each phase of the review (i.e., screening, eligibility, and inclusion in meta-analysis)	✓	☐	134-157
Data collection process	11c	Describe planned method of extracting data from reports (e.g., piloting forms, done independently, in duplicate), any processes for obtaining and confirming data from investigators	✓	☐	149-165
**Data items**	12	List and define all variables for which data will be sought (e.g., PICO items, funding sources), any pre-planned data assumptions and simplifications	✓	☐	209-228
**Outcomes and prioritization**	13	List and define all outcomes for which data will be sought, including prioritization of main and additional outcomes, with rationale	✓	☐	182-208
**Risk of bias in individual studies**	14	Describe anticipated methods for assessing risk of bias of individual studies, including whether this will be done at the outcome or study level, or both; state how this information will be used in data synthesis	✓	☐	295-309
DATA					
**Synthesis**	15a	Describe criteria under which study data will be quantitatively synthesized	✓	☐	182-228
	15b	If data are appropriate for quantitative synthesis, describe planned summary measures, methods of handling data, and methods of combining data from studies, including any planned exploration of consistency (e.g., I^2, Kendall's tau)	✓	☐	229-291
	15c	Describe any proposed additional analyses (e.g., sensitivity or subgroup analyses, meta-regression)	✓	☐	292-300
	15d	If quantitative synthesis is not appropriate, describe the type of summary planned	✓	☐	307-309

For peer review only

BioMed Central
The Open Access Publisher

Section/topic	#	Checklist item	Information reported		Line number(s)
			Yes	No	
**Meta-bias(es)**	16	Specify any planned assessment of meta-bias(es) (e.g., publication bias across studies, selective reporting within studies)	✓	☐	301-309
**Confidence in cumulative evidence**	17	Describe how the strength of the body of evidence will be assessed (e.g., GRADE)	✓	☐	302-303

For peer review only

BioMed Central
The Open Access Publisher
